# Comparative Lipid-Lowering/Increasing Efficacy of 7 Statins in Patients with Dyslipidemia, Cardiovascular Diseases, or Diabetes Mellitus: Systematic Review and Network Meta-Analyses of 50 Randomized Controlled Trials

**DOI:** 10.1155/2020/3987065

**Published:** 2020-04-23

**Authors:** Xiaodan Zhang, Lu Xing, Xiaona Jia, Xiaocong Pang, Qian Xiang, Xia Zhao, Lingyue Ma, Zhiyan Liu, Kun Hu, Zhe Wang, Yimin Cui

**Affiliations:** ^1^Department of Pharmacy, Base for Clinical Trial, Peking University First Hospital, Beijing 100034, China; ^2^Department of Pharmacy, China Pharmaceutical University, Nanjing 210000, China; ^3^Department of Pharmacy Administration and Clinical Pharmacy, School of Pharmaceutical Sciences, Peking University Health Science Center, Beijing 100191, China

## Abstract

**Objective:**

The drug efficacy may differ among different statins, and evidence from head-to-head comparisons is sparse and inconsistent. The study is aimed at comparing the lipid-lowering/increasing effects of 7 different statins in patients with dyslipidemia, cardiovascular diseases, or diabetes mellitus by conducting systematic review and network meta-analyses (NMA) of the lipid changes after certain statins' use.

**Methods:**

In this study, we searched four electronic databases for randomized controlled trials (RCTs) published through February 25, 2020, comparing the lipid-lowering efficacy of no less than two of the included statins (or statin vs. placebo). Three reviewers independently extracted data in duplicate. Firstly, mixed treatment overall comparison analyses, in the form of frequentist NMAs, were conducted using STATA 15.0 software. Then, subgroup analyses were conducted according to different baseline diseases. At last, sensitivity analyses were conducted according to age and follow-up duration. The trial was registered with PROSPERO (number CRD42018108799).

**Results:**

As a result, seven statin monotherapy treatments in 50 studies (51956 participants) were used for the analyses. The statins included simvastatin (SIM), fluvastatin (FLU), atorvastatin (ATO), rosuvastatin (ROS), lovastatin (LOV), pravastatin (PRA), and pitavastatin (PIT). In terms of LDL-C lowering, rosuvastatin ranked 1^st^ with a surface under cumulated ranking (SUCRA) value of 93.1%. The comparative treatment efficacy for LDL-C lowering was ROS>ATO>PIT>SIM>PRA>FLU>LOV>PLA. All of the other ranking and NMA results were reported in SUCRA plots and league tables.

**Conclusions:**

According to the NMAs, it can be concluded that rosuvastatin ranked 1^st^ in LDL-C, ApoB-lowering efficacy and ApoA1-increasing efficacy. Lovastatin ranked 1^st^ in TC- and TG-lowering efficacy, and fluvastatin ranked 1^st^ in HDL-C-increasing efficacy. The results should be interpreted with caution due to some limitations in our review. However, they can provide references and evidence-based foundation for drug selection in both statin monotherapies and statin combination therapies.

## 1. Introduction

Coronary heart disease (CHD) is the leading cause of death in most countries, with a high prevalence currently driven by dual epidemics of obesity and diabetes [1]. Statins are the hypolipidemic treatment of choice for hyperlipidemia with a confirmed atherosclerotic cardiovascular disease (ASCVD) protective effect, proven even in normolipemic patients [2]. Statin drugs are the most effective, evidence-based agents to prevent and treat this disease. Statins have a central role in management and are advised in all published guidelines [1]. Currently, dyslipidemia treatment is based on individualized risk factor assessment. The 2018 AHA/ACC/AACVPR/AAPA/ABC/ACPM/ADA/AGS/APhAHDCDT_3987065/ASPC/NLA/PCNA Guideline on the Management of Blood Cholesterol [3] recommends the use of statins based on risk factors for cardiovascular disease, rather than low-density lipoprotein (LDL) level targets that were formerly used to guide statin intensity according to the Third Report of the National Cholesterol Education Program Expert Panel on Detection, Evaluation, and Treatment of High Blood Cholesterol in Adults (ATP III) dyslipidemia guideline [4].

Nowadays, with the emergence of new preparations and therapeutics, as well as the appearance of some adverse reactions and tolerance phenomenon of statins in their applications, the statin monotherapies have been questioned [1, 2, 5]. Nonstatin therapy has gradually entered the field of vision [6]. However, in the clinical practice, evidence such as RCTs, guidelines, and recommendations for these nonstatin therapies are very limited, which provides little evidence-based efficacy support for clinicians to use only nonstatin therapies in the treatment of dyslipidemia. Therefore, at present, statins are still one of the main drugs for the treatment of hyperlipidemia, especially in combination with other drugs. Absolute nonstatin therapies should only be considered in high-risk patients who have a suboptimal response to statins and/or are intolerant to statin therapy [6].

When it comes to choosing one statin treatment among multiple alternatives, scientific evidence is particularly important. However, existing evidence is insufficient to inform prescribing decisions. While traditional meta-analyses synthesize existing RCT data and compare the efficacy between two statin treatments, network meta-analysis allows for the combination of direct and indirect evidences from randomized trials, facilitating the comparison of all kinds of statins even when they are not directly compared with each other in clinical trials [7].

To date, some statin-related studies have focused on the comparison between statin combination therapies with statin monotherapies [8, 9], and others (mainly network meta-analyses) have focused on the comparative tolerability or comparative effects among different statins [10, 11]; however, the outcome indicators were mainly the occurrence and outcome of relevant diseases. In 2014, Naci et al. published a network meta-analysis using the absolute value of lipid as the primary efficacy indicator of different statins [12]. Similar to the method used in this article, the change values of lipids were chosen as the primary endpoint of our network meta-analyses. The direct lipid-lowering/increasing effects of the 7 statins were compared in this study, providing a reference and evidence-based foundation for drug selection in both statin monotherapies and statin combination therapies.

## 2. Materials and Methods

This paper conforms with the PRISMA-NMA guidance [13]. The trial was registered with PROSPERO (number CRD42018108799) [14].

### 2.1. Data Sources and Searches

A systematic literature review of Cochrane Library, EMBASE, PubMed, and Web of Science electronic databases was performed to identify RCTs comparing the lipid-lowering/increasing effect of no less than two types of the included statins or the effect of placebo and no less than one type of the included statin. Articles published through February 25, 2020, were searched using the following keyword combination strategy: lovastatin (All Fields) OR pravastatin (All Fields) OR simvastatin (All Fields) OR fluvastatin (All Fields) OR atorvastatin (All Fields) OR rosuvastatin (All Fields) OR pitavastatin (All Fields) OR statins (All Fields) AND randomized controlled trial (All Fields). A complete detailed search strategy is included in Appendix S1. EndNote software version X8 was used throughout the literature search and screening process.

### 2.2. Study Selection

The literature search was independently conducted by three authors (XZ, LX, and XJ); in cases of disagreement, a consensus was reached through group discussion. A study was eligible for inclusion if the following criteria were met: (a) a RCT where the random methods, control groups, and blind methods were all included; (b) the study comparing the lipid-lowering efficacy of more than two included statins or placebo with one of the included statins; (c) therapeutic doses of the statins which were administered in the study; and (d) the absolute value change of one of the following six indicators after drug administration which could be directly extracted or calculated from the outcomes of the study: low-density lipoprotein cholesterol (LDL-C), high-density lipoprotein cholesterol (HDL-C), total cholesterol (TC), triglyceride (TG), Human Apolipoprotein A-1 (ApoA1), and Human Apolipoprotein B (ApoB).

Potentially relevant papers and abstracts were obtained, and the full-text editions were reviewed for inclusion. Studies conducted in healthy volunteers or in patients with diseases other than dyslipidemia, cardiovascular diseases, or diabetes mellitus were excluded. Studies published in languages other than English were excluded.

### 2.3. Data Extraction and Quality Assessment

An electronic data abstraction form was used to record basic data, including the first author's name, publication year, number of subjects, ethnicity, subject status (disease type), drug usage/follow-up duration, and outcomes.

The Cochrane Risk of Bias tool was used to assess the methodological quality of the eligible trails [15]. We scored the chosen articles while extracting data, and RevMan 5.3 was used to generate the literature quality assessment table. Any incongruence between the 3 investigators (XZ, LX, and XJ) was reassessed and discussed until a consensus was reached.

Outcome data, the absolute mean changes, standard deviation (SD) of the lipids after treatment, and *n* (number of patients in a certain group), were mostly calculated according to the baseline and endpoint lipid data in the articles. The mean change values were calculated by subtracting the mean of the endpoints from the mean of the baseline. The calculation method of SD was adopted from Cochrane Handbook version 5.1.0 [16]. The included outcomes were absolute change values of LDL-C, HDL-C, TC, TG, ApoA1, and ApoB. Original data were collected in the form of “mean, SD, and *n*,” except for five studies [17–21], in which the original data for TG were presented in the form of “median, quartiles, and *n*.” For these, the mean and SD were estimated using the calculation method described in Wan et al.'s article [22].

In addition, the units of the outcome indicators were unified by unit conversion for the four outcomes (LDL-C, HDL-C, TC, and TG), while ApoB and ApoA1 did not use unit conversion. In this study, we uniformly used mg·dl^−1^ as the unit of measurement. When the unit provided in the original text was mmol·l^−1^, we multiplied the original data by a certain conversion coefficient and converted it to mg·dl^−1^ as the unit. The methods for unit conversion are shown in AppendixS2.

In our overall NMAs, a method of mixing different dosage groups was adopted. The overall NMAs were conducted only separated by different statin treatments, not by different dosage groups, because 10 of the included studies did not use a fixed drug administration dose, preventing their data from being grouped by different drug dosages. When there were two or more dosage groups for the same statin treatment in one study, we first separately calculated the mean change values, SDs, and *n* (sample sizes) of the patients in different dosage groups according to the method described above, and then we merged these dosage groups using the method introduced in the Cochrane Handbook version 5.1.0 [23]. Six of the included studies used this method to merge two dosage groups of the same statin treatment [19, 24–28]. As a result, in each study, different dosage groups of the same statin (if there were no less than two dosage groups) were eventually processed into a single experimental group for final network meta-analysis.

Since our NMA included patients of different types of diseases (dyslipidemia, cardiovascular diseases, or diabetes mellitus), after conducting the overall NMA, we also conducted subgroup analyses according to different baseline diseases of the patients.

At last, sensitivity analyses were conducted according to age and follow-up duration.

### 2.4. Data Synthesis and Analysis

We constructed the network meta-analyses by combining direct and indirect evidence. Frequentist NMA was conducted using the network suite and other network-related commands in STATA 15.0 [29–31]. STATA was also used in the drawing of Network Plots of Network Meta. Global and local inconsistency tests were conducted. Global Wald tests for inconsistency were performed [32, 33]. Local inconsistency was explored by a node-splitting method [33, 34]. Visual inspection of the funnel plots was conducted separately for the 6 outcomes and used to assess publication bias. In addition, to rank the lipid-lowering/increasing effects of treatments, the surface under the cumulative ranking (SUCRA) was used to summarize the probability values. The SUCRA value was 100% for optimal treatment and 0% for worst treatment [32]. League tables were produced for the 6 outcomes, showing the mixed evidence reported results of pair-wise comparisons among different treatments [35].

Subgroup analyses were conducted according to different baseline diseases. Sensitivity analyses were conducted according to age and follow-up duration.

All data were processed through Review Manager (version 5.3), STATA software (version 15.0), or Microsoft Excel 2016.

## 3. Results

### 3.1. Study Characteristics

**Figure 1:**
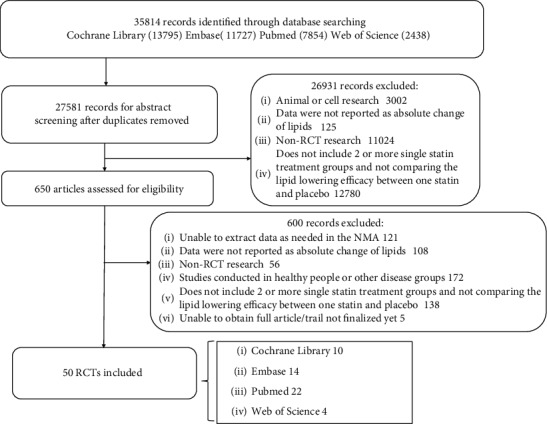
Summary of the article search and selection process (flow chart). RCTs: randomized controlled trials.

The study selection process is presented in Figure 1. The bibliographic search retrieved 35814 citations, and after removing duplicates, we reviewed the remaining 27581 articles in the form of a title and an abstract; 650 citations remained after the title and abstract screening. Eventually, after full-text screening, there were 50 studies eligible for the NMA [17–21, 24–28, 36–75], including 51956 participants. The general characteristics of the included studies are summarized in Table 1. The baseline values of the biochemical parameters in all the included studies are shown in Table 2.

**Figure 2:**
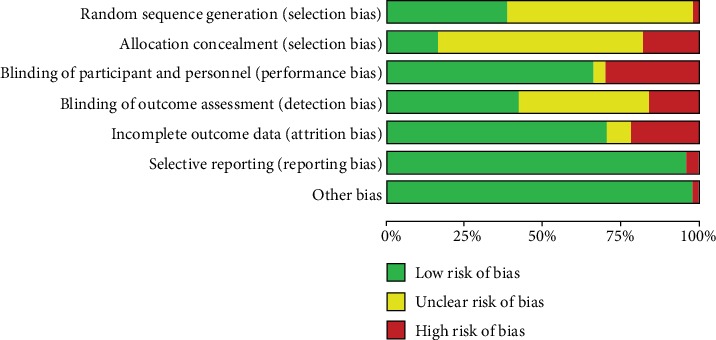
Risk of bias graph: review of authors' judgements about each risk of bias item presented as percentages across all included studies.

**Figure 3:**
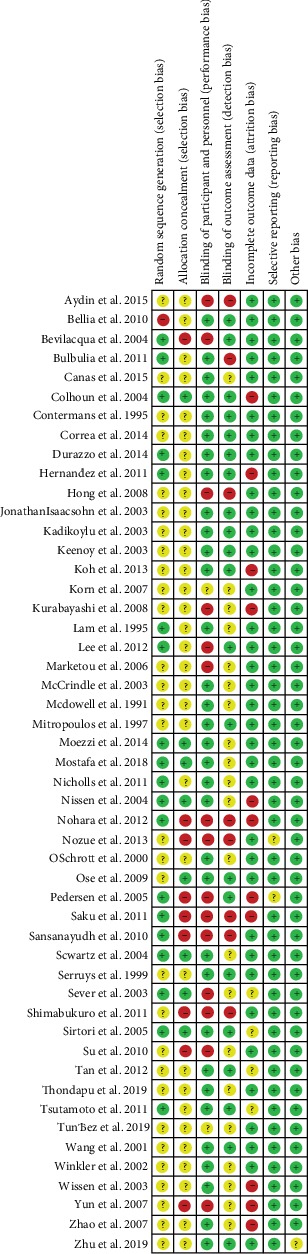
Risk of bias summary: review of authors' judgements about each risk of bias item for each included study.

Reflecting methodological quality of the included studies, the Cochrane Risk of bias tool was used, the risk of bias graph is shown in Figure 2, and the risk of bias summary is shown in Figure 3. As we can see from Table 1, all of the included patients had dyslipidemia, cardiovascular diseases, or diabetes mellitus. Treatment groups covered all of the seven statin treatments and placebo, and the study populations varied from Americans, British people, Italians, Brazilians, Greek, German to Chinese, Japanese, Koreans etc. The included population groups were diversified. As we can see from Table 2, the baseline values of LDL-C, HDL-C, TC, TG, ApoA1, and ApoB are very close, basically distributed at the same level, and they can be combined for analysis in an integrated NMA.

### 3.2. Results of the Overall Network Meta-Analyses

**Figure 4:**
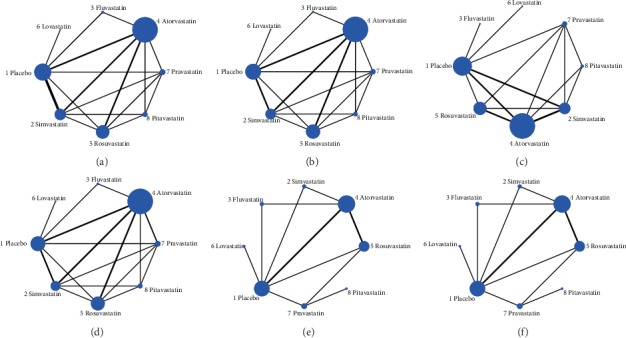
Network plots of eligible comparisons for (a) LDL-C change value, (b) HDL-C change value, (c) TC change value, (d) TG change value, (e) ApoA1 change value, and (f) ApoB change value among placebo and different statin treatments. The size of the nodes is weighted according to the number of trials available for each treatment. The treatments for which direct comparisons were available are linked with a line. The thickness of the line corresponds to the inverse variance of the direct comparisons which is a proxy for the sample size.

50, 45, 43, 40, 15, and 15 studies were separately included in the NMA of the following outcomes: LDL-C, HDL-C, TC, TG, ApoA1, and ApoB. The Network plots are shown in Figures 4(a) – 4(f). The nodes represent the individual drugs; lines represent direct comparisons using clinical trials; the thickness of lines represents the number of available clinical trials.

The results of global inconsistency tests are shown in Table 3, where the P values are listed for each outcome. If the *P* value is larger than 0.05, then the inconsistency model is not significant and the data can be analyzed using a consistency model. As is shown in the table, the *P* values are larger than 0.05, except the *P* value in TG NMA, indicating that there is inconsistency between direct and indirect evidences in the NMA for the TG mean change value.

In addition, local inconsistency was tested using a node-splitting method. The node-splitting models revealed statistically significant inconsistency between direct and indirect evidences in the following comparisons in Table 4.

**Figure 5:**
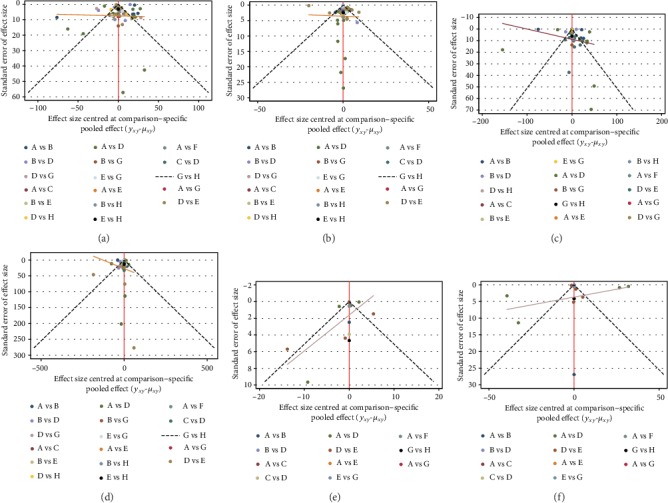
Network funnel plots of (a) LDL-C change value, (b) HDL-C change value, (c) TC change value, (d) TG change value, (e) ApoA1 change value, and (f) ApoB change value among placebo and different statin treatments (A: placebo, B: simvastatin, C: fluvastatin, D: atorvastatin, E: rosuvastatin, F: lovastatin, G: pravastatin, and H: pitavastatin).

Funnel plots were performed to examine publication bias. No obvious publication bias was observed for the 50 studies. The funnel plots are shown in Figure 5.

**Figure 6:**
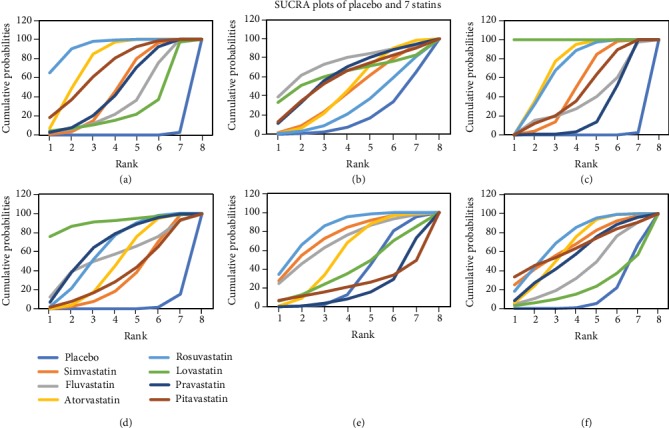
The cumulative rank diagram of the estimated probability among placebo and the 7 compared statins in (a) LDL-C network meta-analysis, (b) HDL-C network meta-analysis, (c) TC network meta-analysis, (d) TG network meta-analysis, (e) ApoA1 network meta-analysis, and (f) ApoB network meta-analysis.

To rank the serum LDL-C-, TC-, TG-, and ApoB-lowering effects of the statins, the surface under the cumulative ranking (SUCRA) was used to summarize the probability values. The HDL-C- and ApoA1-increasing efficacies of the statins were also ranked using this method. The SUCRA value was 100% for the optimal treatment and 0% for the worst treatment. Superposed SUCRA plots are shown in Figure 6, and the SUCRA values for each treatment in 6 different outcomes are shown in Table 5. Combining the results in the plots and in the table, we can conclude that the comparative treatment efficacy for LDL-C lowering is ROS>ATO>PIT>SIM>PRA>FLU>LOV>PLA; the comparative efficacy in HDL-C increasing was FLU>LOV>PRA>PIT>ATO>SIM>ROS>PLA; the comparative TC lowering efficacy was LOV>ATO>ROS>SIM>PIT>FLU>PRA>PLA; the comparative TG lowering efficacy was LOV>PRA>ROS>FLU>ATO>PIT>SIM>PLA; their efficacy on ApoA1 increasing exhibited ROS>SIM>FLU>ATO>LOV>PLA>PIT>PRA; and their efficacy on ApoB-lowering exhibited ROS>SIM>ATO>PIT>PRA>FLU>LOV>PLA.

The league tables for the 6 NMAs were also produced, the league table for LDL-C NMA is shown in Table 6, and the league tables for the other 5 outcomes can be found in AppendixS3. Estimates are presented by the mean difference with 95% confidence interval (CI) in parentheses. For the NMAs of LDL-C, TC, TG, and ApoB, mean differences below 0 suggest that the treatment listed in the upper row is superior, and mean differences above 0 suggest that the treatment listed in the left column is superior. For the NMAs of HDL-C and ApoA1, mean differences above 0 suggest that the treatment listed in the upper row is superior, and mean differences below 0 suggest that the treatment listed in the left column is superior.

### 3.3. Results of the Subgroup NMAs

The global and local inconsistency tests of the overall NMAs exhibited inconsistency in several comparisons for the 6 outcomes. The subgroup NMAs were conducted to find out if the inconsistency was originated from the different diseases of the patient groups.

Patients included in the overall NMAs were grouped according to their baseline disease. We divided the patients into three groups: patients with cardiovascular diseases other than simple dyslipidemia (Group 1), dyslipidemia (Group 2), and diabetes mellitus (Group 3). In the subgroup NMAs for LDL-C, each group included 19, 22, and 9 studies, respectively. Frequentist NMAs were separately conducted for the 3 groups for the 6 outcomes. The global and local inconsistency test results are shown in Tables 7 and 8.

As is shown in the tables, the original global inconsistency revealed in the overall NMA for TG was not eliminated by conducting subgroup analyses. Furthermore, for outcomes ApoA1 and ApoB, there were too few articles included in the subgroup NMAs in which data contain no potential source of heterogeneity, and the global and local inconsistency tests could not be conducted.

### 3.4. Results of the Sensitivity Analyses

Sensitivity analyses were conducted according to age, follow-up duration, and drug dosage. These analyses were conducted to see if the differences in patients' age, studies' follow-up duration, or studies' drug dosage have contributed to the inconsistency in the overall NMAs. Of the six lipid outcomes, only the overall analysis of the TG-lowering effect revealed inconsistency between direct and indirect evidences. Therefore, we have conducted sensitivity analyses only for this outcome.

The method for age sensitivity analysis was to exclude studies on patients under the age of 18 and conduct NMA for the TG mean change value with the remaining studies. Two studies were excluded because the patients were children or adolescents, and the other 8 studies were also excluded because the age range of the included subjects was unknown. The *P* value of the global inconsistency test after study removal was 0.0000, which still indicates inconsistency between direct and indirect evidences.

The method for follow-up duration sensitivity analysis was to divide the studies into 4 groups: (1) studies with follow-up durations of less than 3 months (including 3 months), (2) studies with follow-up durations of 3months to 1 year (including 1 year), (3) studies with follow-up durations of 1-2 years (including 2 years), and (4) studies with follow-up durations of more than 2 years. The global inconsistency tests were conducted separately for these grouped NMAs for TG outcome. The results are shown in Table 9. As is shown in the table, Group 1 included 13 studies and still revealed inconsistency between direct and indirect evidences, while Group 2 included 7 studies and showed no inconsistency. The difference in follow-up durations might have contributed to the inconsistency in the overall NMAs to some extent.

## 4. Discussion

In this study, we conducted 6 network meta-analyses for different outcomes in lipid change. The lipid change value was used as the comparison outcome indicator for each of the NMAs. According to the SUCRA results and league tables, ranks of the 7 statins in terms of LDL-C lowering, HDL-C increasing, TC lowering, TG lowering, TC lowering, ApoA1 increasing, and ApoB lowering were concluded, respectively. Generally speaking, rosuvastatin and atorvastatin exhibited rather great efficacy in regulating serum lipids; this has especially confirmed the abundant use of atorvastatin in clinical applications [4].

Based on previous literature, rosuvastatin and atorvastatin are traditionally high-potency statins, which might have the potential in leading to better clinical outcomes than low-potency statins such as pravastatin, simvastatin, fluvastatin, and lovastatin [76]. Three generations of statins have been introduced before [77]: the first generation statins, lovastatin, pravastatin, and fluvastatin, were introduced in the USA in the late 1980s and 1990s; they represent the class members with the lowest potency; the second generation statins, atorvastatin and simvastatin, have significantly improved efficacy in reducing LDL-C levels compared to the earlier statins; finally, there is a single commercially available drug in the third, high-potency generation of statins, rosuvastatin. Three unique chemical characteristics of rosuvastatin provide enhanced potency against HMG-CoA reductase.

The SUCRA rank results presented in our research are very close to previous findings [77], especially in terms of the efficacy rank of lowering LDL-C, TC, and TG and increasing HDL-C. Rosuvastatin and atorvastatin ranked No. 1 and No. 2 in lowering LDL-C, which is consistent with previous findings. In terms of the efficacy of increasing HDL-C, although the ranking was FLU>LOV>PRA>PIT>ATO>SIM>ROS>PLA; not exactly like the results in previous studies, the SUCRA values of these statins were very close to our result (shown in Figure 6). The results of lowering the TG and TC efficacy rankings are generally consistent with previous studies. It is worth noting that as the first generation of statin, lovastatin ranked first in both TG and TC lowering rankings, which is not the same as we expected. We noticed that only one single study included the use of lovastatin in one group [73]. In this study, the adopted dose of lovastatin was 20-60 mg, relatively larger compared to a particular dose used on patients with simple dyslipidemia. Also, this lovastatin study had a rather long follow-up time of 2 years. These might be the reasons why lovastatin ranked first in both TG and TC lowering rankings.

As we can see from the results, rosuvastatin and atorvastatin have a high efficacy of lowering LDL-C and have a relatively good performance in regulating other blood lipid levels. They are also effective and widely used in clinical applications for regulating serum lipids and treating cardiovascular diseases.

The results of our review should be interpreted with caution in view of the following limitations. First of all, the follow-up periods of the included studies were between 14 days and 5 years. This variation will have an effect in reporting outcome measures using lipid mean change differences. Second, the doses of statins used in the eligible studies were not unified. We included all the RCTs using therapeutic doses of stains, because 10 of the included studies did not have a fixed statin dosage and their data cannot be grouped according to a certain dosage. This disunity of the drug doses might lead to bias in the network meta-analyses. In addition, some of the mean change and SD of lipids data were estimated according to the medians and quartiles collected from original articles, which might bring bias and inaccuracy in the data. This might also be one of the causes responsible for the inconsistency between direct and indirect evidences in several comparisons (see Table 4).

## 5. Conclusions

Rosuvastatin ranked 1st in LDL-C- and ApoB-lowering efficacy and ApoA1-increasing efficacy. Lovastatin ranked 1st in TC- and TG-lowering efficacy, and fluvastatin ranked 1st in HDL-C-increasing efficacy. The results should be interpreted with caution due to some limitations in our review. However, they can still provide some references and evidence-based foundation for drug selection in clinical application.

## Figures and Tables

**Table 1:** General characteristics of studies included in the network meta-analyses.

Study	Group (treatment)	No. of subjects	Mean age	Disease status	Country	Population	Follow-up duration	Outcomes
Zhu et al. [36]	ATO (20 mg) PLA	86	65.78 ± 6.77	Ischemic stroke	China	Chinese	6 months	LDL-C, TC, TG
Tunçez et al. [37]	ATO (80 mg) ROS (40 mg)	63	57.67 ± 9.35 (ATO) 58.30 ± 11.98 (ROS)	Acute myocardial infarction	Turkey	Turk	4 weeks	LDL-C, HDL-C, TC
Thondapu et al. [38]	ATO (20 mg) ROS (10 mg)	43	54.2 (ATO) 57.5 (ROS)	De novo coronary artery disease	USA, Japan, and Korea	UNK	1 year	LDL-C, HDL-C, TC, TG
Mostafa et al. [17]	ATO (80 mg) ROS (40 mg)	100	54.6 ± 9 (ATO) 54.9 ± 8.4 (ROS)	Acute coronary syndrome/dyslipidemia	Arab Republic of Egypt	Egyptians	1 month	LDL-C, HDL-C, TC, TG
Zhao and Peng 2017 [24]	ATO (10 mg) ROS (5 mg, 10 mg)	414	59.5 ± 9.51	Hypercholesterolemia	China	Chinese	6 weeks	LDL-C, HDL-C, TC, TG, ApoB
Canas et al. [39]	PLA ATO	38	15 ± 0.3	Type 1 diabetes	USA	American	6 months	LDL-C, HDL-C, TC, TG
Aydin et al. [40]	ATO (80 mg) ROS (20 mg)	120	58 ± 11	ST elevation myocardial infarction	Turkey	Turk	40 weeks	LDL-C, HDL-C, TC, TG, ApoB, ApoA1
Moezzi et al. [18]	PLA SIM (40 mg)	77	20-88	Dyslipidemia	Iran	Iranian	1 month	LDL-C, HDL-C, TC
Correa et al. [41]	SIM (40 mg) PLA	79	18-70	Hypertension	Brazil	Brazilian	6 months	LDL-C
Koh et al. [42]	PLA ROS (10 mg) PRA (40 mg)	158	UNK	Hypercholesterolemia	Korea	Korean	2 months	LDL-C, HDL-C, TC, TG, ApoB, ApoA1
Nozue et al. [43]	PIT (4 mg) PRA (20 mg)	164	66 ± 9 67 ± 10	Coronary artery disease	Japan	Japanese	8 months	LDL-C, HDL-C, TC, TG, ApoB, ApoA1
Nohara et al. [44]	ROS (5 mg) PRA (10 mg)	298	Adult	Carotid intima-media thickness	Japan	Japanese	24 months	LDL-C, HDL-C, TG
Lee et al. [45]	ATO (20 mg) ROS (10 mg)	271	≧18 years old	Mild coronary atherosclerotic plaques	Korea	Korean	6 months	LDL-C, HDL-C, TC, TG
Nicholls et al. [46]	ATO (80 mg) ROS (40 mg)	1578	18-75	Coronary disease	USA	American	UNK (endpoint time was during treatment)	LDL-C, HDL-C, TC, ApoB, ApoA1
Saku et al. [47]	ATO (10 mg) ROS (2.5 mg) PIT (2 mg)	228	25-75	Hypercholesterolemia	Japan	Japanese	16 weeks	LDL-C, HDL-C, TG
Hernández et al. [19]	PLA ATO (10/40 mg)	62	45-75	Hypercholesterolemia	Spain	Spanish	3 months	LDL-C, HDL-C, TC, TG
Tsutamoto et al. [48]	ROS (2.5 mg) ATO (5 mg)	63	60.6 ± 10.9 59.8 ± 8.8	Cardiac sympathetic nerve activity in nondiabetic patient with dilated cardiomyopathy	Japan	Japanese	6 months	LDL-C, HDL-C, TC, TG
Shimabukuro et al. [49]	PIT (2 mg) ATO (10 mg)	31	30–79	Type 2 diabetes mellitus	Japan	Japanese	6 months	LDL-C, HDL-C, TC, TG
Bulbulia et al. [50]	PLA SIM (40 mg)	20536	40-80	High risk of vascular	Britain	British	3-5 years	LDL-C, TC
Sansanayudh et al. [51]	PIT (1 mg) ATO (10 mg)	100	≧18	Hypercholesterolemia	Thailand	Thai	8 weeks	LDL-C, HDL-C, TC, TG
Bellia et al. [52]	SIM (20 mg) ROS (20 mg)	29	55 ± 3	Type 2 diabetes	Italy	Italian	4 weeks	LDL-C, HDL-C, TC, TG
Su et al. [53]	SIM (40 mg) ATO (10 mg)	151	51–72	Type 2 diabetes mellitus	China	Chinese	12weeks	LDL-C, HDL-C, TC, TG
Ose et al. [25]	PIT (2 mg, 4 mg) SIM (20 mg, 40 mg)	857	18–75	Hypercholesterolemia or dyslipidemia	Russia, Norway, UK, Finland, Italy	Multiple groups	12 months	LDL-C
Kurabayashi et al. [54]	ATO (10 mg) ROS (5 mg)	405	≧20	Hypercholesterolemia	Japan	Japanese	8 weeks	LDL-C, HDL-C, TC, TG
Young et al. [55]	ATO (40 mg) ROS (20 mg)	30	60 ± 8 62 ± 9	Coronary stenosis	Korea	Korean	1 year	LDL-C, HDL-C, TC, TG
Kyeong et al. [56]	ATO (20 mg) ROS (10 mg)	117	63.5 ± 11.67 63.4 ± 10.88	Acute coronary syndrome	Korea	Korean	40 weeks	LDL-C, HDL-C, TC, TG, ApoB, ApoA1
Kom et al. [57]	PLA ATO (40 mg)	24	35-60	Hypercholesterolemia	Germany	German	6 weeks	LDL-C, HDL-C, TC
Marketou et al. [58]	SIM (40 mg) ATO (40 mg)	88	35-70	Hyperlipidemia	Greece	Greek	3 weeks	LDL-C, HDL-C, TG
Pedersen et al. [59]	SIM (20 mg) ATO (80 mg)	8888	≦80	Myocardial infarction	Europe	European	5 years	LDL-C, HDL-C, TC, TG, ApoB, ApoA1
Sirtori et al. [60]	ATO (10 mg) PRA (20 mg)	86	UNK	Hyperlipidemia	Italy	Italian	12 weeks	LDL-C
Nissen et al. [61]	PRA (40 mg) ATO (80 mg)	654	30-75	Coronary atherosclerosis	USA	American	18 months	LDL-C, HDL-C, TC, TG
Durazzo et al. [20]	PLA ATO (20 mg)	100	UNK	After vascular surgery	Brazil	Brazilian	45 days	LDL-C, HDL-C, TC, TG
Bevilacqua et al. [62]	FLU (80 mg) ATO (20 mg)	100	45 to 71	Type 2 diabetes mellitus	Italy	Italian	3 months	LDL-C, HDL-C, TG, ApoB, ApoA1
Schwartz et al. [26]	ROS (5 mg, 10 mg) ATO (10 mg)	382	≧18	Hypercholesterolemia coronary heart disease	US Canada	American Canadian	12 weeks	LDL-C, HDL-C, TC, TG, ApoB, ApoA1
Colhoun et al. [63]	PLA ATO (10 mg)	2819	40–75	Type 2 diabetes mellitus	UK Ireland	European Irish	4 years	LDL-C, HDL-C, TC, TG, ApoB, ApoA1
Wissen et al. [64]	SIM (40 mg) ATO (80 mg)	325	UNK	Heterozygous familial hypercholesterolemia	Netherland	Dutch	2 years	LDL-C, HDL-C, TC, TG
Isaacsohn et al. [27]	PLA SIM (20 mg/40 mg/80 mg)	195	18-70	Hypertriglyceridemia	USA	American	6 weeks	LDL-C, HDL-C, TC
McCrindle et al. [65]	ATO (10 mg-20 mg) PLA	187	10-17	Hypercholesterolemia	USA Canada Europe South Africa	American Canadian European African	26 weeks	LDL-C, HDL-C, TC, TG, ApoB, ApoA1
Kadikoylu et al. [66]	ATO (10-20 mg) SIM (10-20 mg)	61	39–74	Primary hypercholesterolemia	USA Europe	American European	24 weeks	LDL-C, HDL-C, TC, TG
Manuel-Y-Keenoy et al. [67]	ATO (40 mg) PLA	24	UNK	Type 1 diabetes	Belgium	European	12 weeks	LDL-C, HDL-C, TC, TG, ApoB, ApoA1
Sever et al. [68]	PLA ATO (10 mg)	10306	40–79	Hypertension	London	British	5 years	LDL-C, HDL-C, TC, TG
Winkler et al. [69]	PLA FLU (80 mg)	89	39–86	Type 2 diabetes mellitus	Germany	German	8 weeks	LDL-C, HDL-C, TC, TG, ApoB, ApoA1
Tan et al. [21]	PLA ATO (10 mg-20 mg)	80	UNK	Type 2 diabetes mellitus	Hong Kong	Chinese	6 months	LDL-C, HDL-C, TC
Wang and Ting [70]	ATO (10 mg) PLA	54	60	Elevated LDL cholesterol	Taiwan	Chinese	8 weeks	LDL-C, HDL-C, TC, TG
Schrott et al. [71]	PLA ATO	22	47-72	Modestly overweight (potential tendency of dyslipidemia)	USA	American	14 days	LDL-C, HDL-C, TC, TG
Serruys et al. [72]	PLA FLU [80 mg (40 mg bid)]	1054	60 ± 9 61 ± 9	After successful coronary balloon angioplasty	Netherlands	Dutch	26 weeks	LDL-C
Mitropoulos et al. [28]	PLA SIM (20 mg, 40 mg)	162	40-75	Coronary heart disease	London	British	2 years	LDL-C, HDL-C, TC, TG
Lam et al. [73]	PLA LOV (20 mg~60 mg)	34	UNK	Hypercholesterolemia	China	Chinese	1 year	LDL-C, HDL-C, TC, TG, ApoB, ApoA1
Contermans et al. [74]	SIM PRA	24	51 ± 8	Hypercholesterolemia	Holland	Dutch	18 weeks	LDL-C, HDL-C, TC, TG
Mcdowell et al. [75]	PLA SIM (10 mg-40 mg)	27	UNK	Primary hypercholesterolemia	Ireland	Irish	12 weeks	LDL-C, HDL-C, TC, TG, ApoB, ApoA1

PLA: placebo; SIM: simvastatin; FLU: fluvastatin; ATO: atorvastatin; ROS: rosuvastatin; LOV: lovastatin; PRA: pravastatin; PIT: pitavastatin; LDL-C: low-density lipoprotein cholesterol; HDL-C: high-density lipoprotein cholesterol; TC: total cholesterol; TG: triglyceride; ApoA1: Human Apolipoprotein A-1; ApoB: Human Apolipoprotein B; UNK: unknown.

**Table 2:** Baseline characteristics of the biochemical values in the included studies.

Study	LDL-C (mg·dl^−1^)	HDL-C (mg·dl^−1^)	TC (mg·dl^−1^)	TG (mg·dl^−1^)	ApoA1	ApoB
Zhu et al. [36]	160.00 ± 11.97 (ATO) 157.91 ± 23.55 (PLA)	NA	222.48 ± 47.67 (ATO) 216.28 ± 60.08 (PLA)	266.39 ± 54.87 (ATO) 270.81 ± 64.61 (PLA)	NA	NA
Tunçez et al. [37]	120.08 ± 27.67 (ATO) 131.69 ± 24.61 (ROS)	36.33 ± 9.76 (ATO) 37.60 ± 10.72 (ROS)	181.64 ± 35.42 (ATO) 206.33 ± 36.00 (ROS)	NA	NA	NA
Thondapu et al. [38]	115 ± 28 (ATO) 100 ± 21 (ROS)	50 ± 12 (ATO) 51 ± 15 (ROS)	203 ± 40 (ATO) 190 ± 44 (ROS)	183 ± 83 (ATO) 245 ± 214 (ROS)	NA	NA
Mostafa et al. [17]	128 ± 45.3 (ATO) 139.1 ± 37.6 (ROS)	36.4 ± 9.5 (ATO) 38.7 ± 13.3 (ROS)	191.7 ± 48.1 (ATO) 199.2 ± 53.2 (ROS)	188 (ATO)^#^ 153 (ROS)^#^	NA	NA
Zhao and Peng [24]	161.97	48.64	245.69	177.11	1.45 mmol·l^−1^	1.20 mmol·l^−1^
Canas et al. [39]	126 ± 5 (PLA) 128 ± 4 (ATO)	63 ± 4 (PLA) 58 ± 3 (ATO)	206 ± 6 (PLA) 203 ± 5 (ATO)	88 ± 13 (PLA) 84 ± 7 (ATO)	4.01 ± 0.13 (PLA) 3.83 ± 0.16 (ATO) mmol·l^−1^	2.4 ± 0.08 (PLA) 2.43 ± 0.10 (ATO) mmol·l^−1^
Aydin et al. [40]	144 ± 25 (ATO) 141 ± 28 (ROS)	38 ± 8 (ATO) 38 ± 9 (ROS)	204 ± 31 (ATO) 201 ± 35 (ROS)	116 ± 72 (ATO) 109 ± 67 (ROS)	118 ± 23 (ATO) 118 ± 26 (ROS) mg·dl^−1^	98 ± 19 (ATO) 99 ± 22 (ROS) mg·dl^−1^
Moezzi et al. [18]	118.38 ± 30.48 (PLA) 131.44 ± 28.46 (SIM)	42.40 ± 11.92 (PLA) 44.08 ± 10.80 (SIM)	193.32 ± 39.65 (PLA) 203.02 ± 36.11 (SIM)	1.24 (PLA)^#^ 1.325 (SIM)^#^	NA	NA
Correa et al. [41]	133.3 ± 30.3 (PLA) 120.8 ± 31.0 (SIM)	52.6 ± 12.1 (PLA) 50.7 ± 11.8 (SIM)	213.4 ± 36.9 (PLA) 198.9 ± 38.8 (SIM)	137.1 ± 61.8 (PLA) 139.7 ± 65.3 (SIM)	NA	NA
Koh et al. [42]	166 ± 4 (PLA) 166 ± 4 (ROS) 165 ± 3 (PRA)	54 ± 1 (PLA) 53 ± 2 (ROS) 51 ± 1 (PRA)	248 ± 4 (PLA) 246 ± 3 (ROS) 241 ± 4 (PRA)	138 ± 10 (PLA) 136 ± 8 (ROS) 136 ± 8 (PRA)	153 ± 2 (PLA) 152 ± 3 (ROS) 151 ± 3 (PRA) mg·dl^−1^	126 ± 3 (PLA) 127 ± 3 (ROS) 128 ± 3 (PRA) mg·dl^−1^
Nozue et al. [43]	123 ± 24 (PIT) 135 ± 35 (PRA)	47 ± 12 (PIT) 46 ± 11 (PRA)	196 ± 31 (PIT) 207 ± 37 (PRA)	128 ± 75 (PIT) 129 ± 56 (PRA)	118 ± 21 (PIT) 118 ± 20 (PRA) mg·dl^−1^	99 ± 19 (PIT) 107 ± 27 (PRA) mg·dl^−1^
Nohara et al. [44]	163.8 ± 30.9 (ROS) 165.1 ± 29.1 (PRA)	54.2 ± 12.1 (ROS) 54.8 ± 13.2 (PRA)	NA	149.6 ± 80.3 (ROS) 136.1 ± 69.8 (PRA)	NA	NA
Lee et al. [45]	110 ± 31 (ATO) 109 ± 31 (ROS)	40 ± 13 (ATO) 40 ± 9 (ROS)	183 ± 36 (ATO) 186 ± 34 (ROS)	165 ± 93 (ATO) 182 ± 121 (ROS)	NA	NA
Nicholls et al. [46]	119.9 ± 28.9 (ATO) 120.0 ± 27.3 (ROS)	44.7 ± 10.7 (ATO) 45.3 ± 11.8 (ROS)	193.5 ± 34.2 (ATO) 193.9 ± 34.1 (ROS)	130 (ATO)^#^ 128 (ROS)^#^	126.2 ± 23.3 (ATO) 128.0 ± 25.2 (ROS) mg·dl^−1^	104.9 ± 21.7 (ATO) 105.4 ± 21.2 (ROS) mg·dl^−1^
Saku et al. [47]	162 ± 24 (ATO) 172 ± 28 (ROS) 164 ± 23 (PIT)	56.7 ± 13.6 (ATO) 57.1 ± 13.4 (ROS) 59.0 ± 14.4 (PIT)	NA	142 ± 70 (ATO) 142 ± 77 (ROS) 132 ± 71 (PIT)	NA	NA
Hernández et al. [19]	168 ± 28 (PLA) 165 ± 33 (ATO)	55 ± 9 (PLA) 56 ± 11 (ATO)	255 ± 33 (PLA) 252 ± 36 (ATO)	125 (PLA)^#^ 128 (ATO)^#^	NA	NA
Tsutamoto et al. [48]	111 ± 28 (ROS) 115 ± 32 (ATO)	43 ± 10 (ROS) 42.6 ± 11 (ATO)	NA	192 ± 80 (ROS) 190 ± 108 (ATO)	NA	NA
Shimabukuro et al. [49]	166.80 ± 19.69 (PIT) 163.32 ± 30.89 (ATO)	51.94 ± 10.85 (PIT) 55.04 ± 10.85 (ATO)	251.94 ± 22.09 (PIT) 255.04 ± 30.62 (ATO)	(PIT) (ATO)	1.35 ± 0.17 (PIT) 1.43 ± 0.10 (ATO) g·l^−1^	1.22 ± 0.10 (PIT) 1.37 ± 0.18 (ATO) g·l^−1^
Bulbulia et al. [50]	131.27 ± 0.39 (PLA) 131.27 ± 0.39 (SIM)	NA	228.68 ± 0.39 (PLA) 228.68 ± 0.39 (SIM)	NA	NA	NA
Sansanayudh et al. [51]	175.99 ± 34.54 (PIT) 172.86 ± 34.53 (ATO)	53.40 ± 15.59 (PIT) 53.92 ± 13.05 (ATO)	258.44 ± 41.25 (PIT) 255.16 ± 40.29 (ATO)	145.22 ± 56.95 (PIT) 141.86 ± 49.08 (ATO)	NA	NA
Bellia et al. [52]	139.77 ± 22.01 (SIM) 133.59 ± 11.97 (ROS)	43.02 ± 7.75 (SIM) 34.11 ± 5.81 (ROS)	205.43 ± 26.74 (SIM) 194.19 ± 17.83 (ROS)	124.79 (SIM) 128.33 (ROS)	NA	NA
Su et al. [53]	128.68 ± 33.33 (SIM) 127.13 ± 24.81 (ATO)	43.80 ± 8.91 (SIM) 44.57 ± 8.14 (ATO)	179.07 ± 18.99 (SIM) 213.57 ± 24.03 (ATO)	151.34 ± 14.16 (SIM) 152.22 ± 15.93 (ATO)	NA	NA
Ose et al. [25]	183.85 (PIT) 184.05 (SIM)	52.06 (PIT) 51.66 (SIM)	267.80 (PIT) 267.69 (SIM)	160.03 (PIT) 160.21 (SIM)	162.56 (PIT) 162.56 (SIM) mg·dl^−1^	160.74 (PIT) 162.64 (SIM) mg·dl^−1^
Kurabayashi et al. [54]	109.3 ± 30.6 (ATO) 102.9 ± 25.1 (ROS)	60.1 ± 15.3 (ATO) 60.9 ± 17.6 (ROS)	192.3 ± 34.8 (ATO) 186.1 ± 28.8 (ROS)	130.9 ± 72.2 (ATO) 128.5 ± 67.4 (ROS)	NA	NA
Young et al. [55]	121 ± 45 (ROS) 127 ± 37 (ATO)	52 ± 7 (ROS) 46 ± 12 (ATO)	180 ± 52 (ROS) 182 ± 45 (ATO)	95 ± 43 (ROS) 84 ± 54 (ATO)	NA	NA
Kyeong et al. [56]	139.1 ± 37.64 (ROS) 137.7 ± 40.92 (ATO)	50.1 ± 13.76 (ROS) 48.8 ± 13.39 (ATO)	198.3 ± 43.24 (ROS) 202.4 ± 45.48 (ATO)	138.3 ± 70.68 (ROS) 140.2 ± 83.99 (ATO)	142.5 ± 28.92 (ROS) 137.9 ± 27.25 (ATO) mg·dl^−1^	102.9 ± 30.49 (ROS) 108.8 ± 29.73 (ATO) mg·dl^−1^
Kom et al. [57]	202 ± 21 (PLA) 231 ± 54 (ATO)	50.6 ± 13.1 (PLA) 59.1 ± 11.6 (ATO)	284 ± 30 (PLA) 320 ± 61 (ATO)	NA	NA	NA
Marketou et al. [58]	178 ± 210 (ATO) 177 ± 210 (SIM)	40 ± 90 (ATO) 42 ± 11 (SIM)	279 ± 240 (ATO) 278 ± 310 (SIM)	227 ± 117 (ATO) 242 ± 880 (SIM)	NA	NA
Pedersen et al. [59]	121.4 ± 0.5 (SIM) 121.6 ± 0.5 (ATO)	46.1 ± 0.2 (SIM) 46.0 ± 0.2 (ATO)	195.9 ± 0.6 (SIM) 196.8 ± 0.6 (ATO)	146.6 ± 1.1 (SIM) 151.1 ± 1.2 (ATO)	1.39 ± 0.01 (SIM) 1.39 ± 0.01 (ATO) g·l^−1^	1.19 ± 0.01 (SIM) 1.19 ± 0.01 (ATO) g·l^−1^
Sirtori et al. [60]	210.9 ± 46.6 (ATO) 225.0 ± 43.9 (PRA)	45.6 ± 13.2 (ATO) 47.6 ± 12.0 (PRA)	305.9 ± 54.1 (ATO) 312.6 ± 43.8 (PRA)	289.1 ± 210.8 (ATO) 237.3 ± 138.2 (PRA)	166.4 ± 28.4 (ATO) 167.1 ± 30.6 (PRA) mg·dl^−1^	153.2 ± 33.5 (ATO) 161.5 ± 29.8 (PRA) mg·dl^−1^
Nissen et al. [61]	150.2 ± 25.9 (PRA) 150.2 ± 27.9 (ATO)	42.9 ± 11.4 (PRA) 42.3 ± 9.9 (ATO)	232.6 ± 34.1 (PRA) 231.8 ± 34.2 (ATO)	197.7 ± 105.6 (PRA) 197.2 ± 95.7 (ATO)	NA	153.0 ± 22.5 (PRA) 152.4 ± 24.3 (ATO) mg·dl^−1^
Durazzo et al. [20]	144.60 ± 32.58 (ATO) 139.65 ± 41.62 (PLA)	44.41 ± 9.37 (ATO) 43.38 ± 13.42 (PLA)	222.74 ± 51.59 (ATO) 214.52 ± 53.25 (PLA)	128 (ATO)^#^ 156.18 (PLA)^#^	NA	NA
Bevilacqua et al. [62]	149 ± 33 (FLU) 141 ± 25 (ATO)	41 ± 7 (FLU) 41 ± 7 (ATO)	NA	437 ± 287 (FLU) 411 ± 271 (ATO)	NA	NA
Schwartz et al. [26]	188 ± 19 (ROS 5 mg) 186 ± 20 (ROS 10 mg) 188 ± 23 (ATO)	46 ± 10 (ROS 5 mg) 47 ± 10 (ROS 10 mg) 47 ± 11 (ATO)	274 ± 26 (ROS 5 mg) 272 ± 24 (ROS 10 mg) 275 ± 27 (ATO)	196 ± 71 (ROS 5 mg) 195 ± 72 (ROS 10 mg) 202 ± 77 (ATO)	143 ± 23 (ROS 5 mg) 143 ± 25 (ROS 10 mg) 142 ± 23 (ATO) mg·dl^−1^	182 ± 21 (ROS 5 mg) 176 ± 20 (ROS 10 mg) 183 ± 22 (ATO) mg·dl^−1^
Colhoun et al. [63]	116.6 ± 27.03 (PLA) 117.4 ± 27.80 (ATO)	55.04 ± 13.18 (PLA) 53.88 ± 12.40 (ATO)	207.4 ± 31.78 (PLA) 207.8 ± 32.17 (ATO)	147.80 (PLA)# 150.45 (ATO)#	1530 ± 294 (PLA) 1530 ± 271 (ATO) mg·l^−1^	1150 ± 241 (PLA) 1170 ± 243 (ATO) mg·l^−1^
Wissen et al. [64]	321.6 ± 78.38 (SIM) 185.7 ± 53.28 (ATO)	44.96 ± 10.85 (SIM) 45.74 ± 12.40 (ATO)	398.1 ± 81.40 (SIM) 387.2 ± 72.48 (ATO)	163.7 ± 118.6 (SIM) 165.5 ± 96.47 (ATO)	NA	NA
Isaacsohn et al. [27]	NA	NA	NA	405 #	NA	NA
McCrindle et al. [65]	218.5 ± 3.47 (ATO) 230.1 ± 6.95 (PLA)	46.12 ± 0.78 (ATO) 46.51 ± 1.55 (PLA)	286.1 ± 3.88 (ATO) 298.8 ± 7.36 (PLA)	102.7 ± 5.31 (ATO) 106.2 ± 7.97 (PLA)	1.25 ± 0.02 (ATO) 1.25 ± 0.03 (PLA) g·l^−1^	1.86 ± 0.03 (ATO) 1.94 ± 0.05 (PLA) g·l^−1^
Kadikoylu et al. [66]	168.5 ± 29.8 (ATO) 172.1 ± 22.5 (SIM)	53.6 ± 9.5 (ATO) 57.5 ± 19.0 (SIM)	263.8 ± 29.9 (ATO) 264.6 ± 23.7 (SIM)	221.5 ± 92.9 (ATO) 191.2 ± 92.3 (SIM)	NA	NA
Manuel-Y-Keenoy et al. [67]	185.7 ± 61.78 (ATO) 139.0 ± 35.91 (PLA)	53.10 ± 11.24 (ATO) 51.16 ± 13.18 (PLA)	263.6 ± 64.34 (ATO) 215.5 ± 39.15 (PLA)	117.7 ± 27.44 (ATO) 125.7 ± 40.71 (PLA)	122 ± 25 (ATO) 116 ± 24 (PLA) mg·dl^−1^	142 ± 37 (ATO) 116 ± 24 (PLA) mg·dl^−1^
Sever et al. [68]	131.3 ± 27.03 (ATO) 131.3 ± 27.03 (PLA)	50.39 ± 15.50 (ATO) 50.39 ± 15.50 (PLA)	213.2 ± 31.01 (ATO) 213.2 ± 31.01 (PLA)	150.5 ± 79.65 (ATO) 150.5 ± 79.65 (PLA)	NA	NA
Winkler et al. [69]	130.1 ± 28.19 (FLU) 238.2 ± 37.84 (PLA)	45.35 ± 12.02 (FLU) 42.25 ± 13.95 (PLA)	245.0 ± 37.98 (FLU) 239.2 ± 37.98 (PLA)	213.3 ± 121.3 (FLU) 215.1 ± 99.12 (PLA)	NA	1.35 ± 0.24 (FLU) 1.36 ± 0.23 (PLA) g·l^−1^
Tan et al. [21]	165.6 ± 20.08 (PLA) 171.8 ± 33.59 (ATO)	43.41 ± 8.91 (PLA) 46.12 ± 8.91 (ATO)	237.2 ± 24.81 (PLA) 246.1 ± 37.98 (ATO)	124.8 (PLA) # 122.1 (ATO) #	NA	NA
Wang and Ting [70]	192.70 ± 20.00 (ATO) 187.29 ± 18.31 (PLA)	45.4 ± 92.1 (ATO) 45.11 ± 10.6 (PLA)	267.0 ± 24.9 (ATO) 260.1 ± 21.9 (PLA)	144.8 ± 45.3 (ATO) 138.5 ± 50.1 (PLA)	NA	NA
Schrott et al. [71]	NA	50 ± 3.5 (PLA) 53 ± 4.8 (ATO)	217 ± 6.3 (PLA) 226 ± 10.5 (ATO)	140 ± 15.5 (PLA) 137 ± 20.1 (ATO)	NA	NA
Serruys et al. [72]	152.9 ± 32.82 (FLU) 152.5 ± 33.59 (PLA)	41.09 ± 10.47 (FLU) 41.86 ± 10.85 (PLA)	222.9 ± 39.15 (FLU) 223.7 ± 39.92 (PLA)	149.6 ± 74.34 (FLU) 141.6 ± 73.46 (PLA)	NA	NA
Mitropoulos et al. [28]	182.6 ± 40.54 (SIM40) 191.1 ± 42.08 (SIM20) 268.0 ± 40.93 (PLA)	44.57 ± 13.18 (SIM40) 46.12 ± 15.12 (SIM20) 44.96 ± 10.47 (PLA)	270.2 ± 43.41 (SIM40) 277.1 ± 54.26 (SIM20) 269.0 ± 41.09 (PLA)	214.2 ± 114.2 (SIM40) 197.4 ± 113.3 (SIM20) 219.5 ± 132.8 (PLA)	NA	NA
Lam et al. [73]	166.0 ± 11.58 (LOV) 158.3 ± 7.72 (PLA)	42.64 ± 1.94 (LOV) 42.64 ± 2.71 (PLA)	255.8 ± 3.88 (LOV) 244.2 ± 3.88 (PLA)	194.7 ± 26.55 (LOV) 256.7 ± 44.25 (PLA)	2.01 ± 0.06 (LOV) 2.02 ± 0.06 (PLA) g·l^−1^	1.55 ± 0.05 (LOV) 1.36 ± 0.05 (PLA) g·l^−1^
Contermans et al. [74]	231.7 ± 32.82 (SIM) 234.0 ± 43.63 (PRA)	42.64 ± 10.47 (SIM) 44.57 ± 4.65 (PRA)	303.9 ± 33.72 (SIM) 307.0 ± 41.86 (PRA)	147.0 ± 52.22 (SIM) 132.8 ± 17.70 (PRA)	NA	NA
Mcdowell et al. [75]	366.8 ± 23.17 (PLA) 351.4 ± 23.17 (SIM)	56.59 ± 3.49 (PLA) 52.71 ± 4.26 (SIM)	453.5 ± 23.26 (PLA) 411.9 ± 23.26 (SIM)	177.0 ± 44.25 (PLA) 230.1 ± 44.25 (SIM)	1.45 ± 0.07 (PLA) 1.41 ± 0.07 (SIM) g·l^−1^	1.69 ± 0.13 (PLA) 1.55 ± 0.11 (SIM) g·l^−1^

PLA: placebo; SIM: simvastatin; FLU: fluvastatin; ATO: atorvastatin; ROS: rosuvastatin; LOV: lovastatin; PRA: pravastatin; PIT: pitavastatin; LDL-C: low-density lipoprotein cholesterol; HDL-C: high-density lipoprotein cholesterol; TC: total cholesterol; TG: triglyceride; ApoA1: Human Apolipoprotein A-1; ApoB: Human Apolipoprotein B. Most data are shown as the “mean ± SD (treatment group).” ^#^Data are shown as the median. NA: not applicable.

**Table 3:** Global inconsistency test results for the 6 different outcomes.

Outcomes	LDL-C	HDL-C	TC	**TG**	ApoA1	ApoB
*P* value	0.8320	0.9886	0.9950	**0.0052**	0.2333	0.8143

**Table 4:** Inconsistency revealed in the node-splitting tests.

Outcomes of the NMAs	Inconsistency was observed between direct and indirect evidences in these comparisons
LDL-C	Placebo vs. lovastatin
HDL-C	Placebo vs. lovastatin
TC	Placebo vs. fluvastatin; placebo vs. lovastatin
TG	Placebo vs. lovastatin
ApoA1	Placebo vs. fluvastatin; fluvastatin vs. atorvastatin
ApoB	None

**Table 5:** SUCRA values of placebo and the 7 statin treatments in NMAs conducted separately for 6 outcomes.

Treatments	LDL-C	HDL-C	TC	TG	ApoA1	ApoB
Placebo	0.4	18.3	0.4	2.3	33.7	13.8
Simvastatin	48.4	44.5	50.0	34.0	75.2	66.2
Fluvastatin	36.4	**74.5**	37.4	56.2	69.2	40.4
Atorvastatin	76.7	47.9	72.4	48.6	56.4	64.5
Rosuvastatin	**93.1**	29.9	69.5	63.1	**82.8**	**72.9**
Lovastatin	27.8	63.3	**100.0**	**91.4**	40.3	21.7
Pravastatin	47.6	62.1	24.5	67.8	18.8	56.6
Pitavastatin	69.5	59.4	45.8	36.6	23.5	63.9

**Table 6:** League table of the LDL-C network meta-analysis results among placebo and 7 statins. (Results with statistical significance are shown in bold.).

	Rosuvastatin	Atorvastatin	Pitavastatin	Simvastatin	Pravastatin	Fluvastatin	Lovastatin	Placebo
Rosuvastatin	Rosuvastatin	5.87 (-5.72, 17.47)	8.26 (-12.46, 28.98)	15.35 (-0.24, 30.95)	16.15 (-2.87, 35.16)	23.03 (-4.12,50.19)	33.67 (-9.49, 76.83)	**72.28 (57.08, 87.48)**
Atorvastatin	-5.87 (-17.47, 5.72)	Atorvastatin	2.38 (-16.71, 21.48)	9.48 (-2.91, 21.88)	10.27 (-7.68, 28.22)	17.16 (-7.82,42.14)	27.79 (-14.15, 69.74)	**66.40 (55.10, 77.71)**
Pitavastatin	-8.26 (-28.98, 12.46)	-2.38 (-21.48, 16.71)	Pitavastatin	7.10 (-12.51, 26.71)	7.89 (-14.86, 30.63)	14.78 (-16.04,45.59)	25.41 (-20.05, 70.87)	**64.02 (43.17, 84.87)**
Simvastatin	-15.35 (-30.95, 0.24)	-9.48 (-21.88, 2.91)	-7.10 (-26.71, 12.51)	Simvastatin	0.79 (-18.99, 20.57)	7.68 (-18.77, 34.12)	18.31 (-24.09, 60.71)	**56.92 (44.05, 69.80)**
Pravastatin	-16.15 (-35.16, 2.87)	-10.27 (-28.22, 7.68)	-7.89 (-30.63, 14.86)	-0.79 (-20.57, 18.99)	Pravastatin	6.89 (-23.21, 36.99)	17.52 (-27.45, 62.49)	**56.13 (36.37, 75.89)**
Fluvastatin	-23.03 (-50.19, 4.12)	-17.16 (-42.14, 7.82)	-14.78 (-45.59, 16.04)	-7.68 (-34.12, 18.77)	-6.89 (-36.99, 23.21)	Fluvastatin	10.64 (-36.38, 57.66)	**49.25 (25.19, 73.31)**
Lovastatin	-33.67 (-76.83, 9.49)	-27.79 (-69.74, 14.15)	-25.41 (-70.87, 20.05)	-18.31 (-60.71, 24.09)	-17.52 (-62.49, 27.45)	-10.64 (-57.66, 36.38)	Lovastatin	38.61 (-1.79, 79.01)
Placebo	**-72.28 (-87.48, -57.08)**	**-66.40 (-77.71, -55.10)**	**-64.02 (-84.87, -43.17)**	**-56.92 (-69.80, -44.05)**	**-56.13 (-75.89, -36.37)**	**-49.25 (-73.31, -25.19)**	-38.61 (-79.01, 1.79)	Placebo

**Table 7:** Global inconsistency test results (*P* values) in NMAs separately conducted for 6 outcomes.

	LDL-C	HDL-C	TC	TG	ApoA1	ApoB
Group 1	**0.0000**	0.8766	0.6987	0.9497	NA^∗∗^	NA^∗∗^
Group 2	0.9991	0.9526	0.8748	**0.0000**	NA^∗∗^	NA^∗∗^
Group 3	0.3887	0.1622	NA^∗^	0.1788	NA^∗∗^	NA^∗∗^

NA^∗^: data contain no potential source of inconsistency; NA^∗∗^: too few articles included in the subgroup analysis, data contain no potential source of heterogeneity.

**Table 8:** Inconsistency revealed in the subgroup NMA node-splitting tests.

Outcomes of the NMAs	Groups	Inconsistency was observed between direct and indirect evidences in these comparisons
LDL-C	Group 1	Placebo vs. fluvastatin; atorvastatin vs. rosuvastatin; atorvastatin vs. lovastatin; rosuvastatin vs. lovastatin
	Group 2	Placebo vs. rosuvastatin
	Group 3	None (no indirect comparison involved)

HDL-C	Group 1	None (no indirect comparison involved)
	Group 2	Placebo vs. rosuvastatin
	Group 3	None (no indirect comparison involved)

TC	Group 1	None (no indirect comparison involved)
	Group 2	None (no indirect comparison involved)
	Group 3	None (no indirect comparison involved)

TG	Group 1	None (no indirect comparison involved)
	Group 2	None (no indirect comparison involved)
	Group 3	None (no indirect comparison involved)

ApoA1	Group 1	NA^∗∗^
	Group 2	NA^∗∗^
	Group 3	NA^∗∗^

ApoB	Group 1	NA^∗∗^
	Group 2	NA^∗∗^
	Group 3	NA^∗∗^

NA^∗∗^: too few articles included in the subgroup analysis, data contain no potential source of heterogeneity.

**Table 9:** Sensitivity analysis results for different follow-up durations. (Global inconsistency test results in NMAs for TG, separately conducted in the 4 follow-up duration groups.).

Groups	Group 1	Group 2	Group 3	Group 4
Number of included studies	13	7	3	3
*P* value	**0.0227**	0.6174	NA^∗∗^	NA^∗∗^

NA^∗∗^: too few articles included in the subgroup analysis, data contain no potential source of heterogeneity.

## Supplementary Materials

Supplementary Materials The Supplementary Material consists of 4 parts. Appendix S1: the complete literature search strategy of the network meta-analysis (NMA). Appendix S2: description of the unit conversion methods used in this study. Appendix S3: league tables of the NMAs conducted for 5 of the outcomes. Appendix S4: the PRISMA checklist of the NMA.

## References

[1] Kones R., Rumana U. (2015). Current treatment of dyslipidemia: a new paradigm for statin drug use and the need for additional therapies. *Drugs*.

[2] Vavlukis M., Vavlukis A. (2018). Adding ezetimibe to statin therapy: latest evidence and clinical implications. *Drugs in Context*.

[3] Grundy S. M., Stone N. J., Bailey A. L. (2018). AHA/ACC/AACVPR/AAPA/ABC/ACPM/ADA/AGS/APhA/ASPC/NLA/PCNA Guideline on the Management of Blood Cholesterol: A Report of the American College of Cardiology/American Heart Association Task Force on Clinical Practice Guidelines. *Circulation*.

[4] Maxwell W. D., Ramsey L. B., Johnson S. G. (2017). Impact of Pharmacogenetics on Efficacy and Safety of Statin Therapy for Dyslipidemia. *Pharmacotherapy: The Journal of Human Pharmacology and Drug Therapy*.

[5] Ansell B. J. (2005). Rationale for combination therapy with statin drugs in the treatment of dyslipidemia. *Current Atherosclerosis Reports*.

[6] Sisson E. M., Pamulapati L., Bucheit J. D., Kelly M. S., Dixon D. L. (2018). Evolving role of non-statin therapy for the management of dyslipidemia and cardiovascular risk reduction: past, present, and future. *Pharmacotherapy: The Journal of Human Pharmacology and Drug Therapy*.

[7] Naci H., Van Valkenhoef G., Higgins J. P., Fleurence R., Ades A. E. (2014). Evidence-based prescribing: combining network meta-analysis with multicriteria decision analysis to choose among multiple drugs. *Circulation. Cardiovascular Quality and Outcomes*.

[8] Choi H. D., Chae S. M. (2018). Comparison of efficacy and safety of combination therapy with statins and omega-3 fatty acids versus statin monotherapy in patients with dyslipidemia. *Medicine*.

[9] Wu N. Q., Guo Y. L., Zhu C. G. (2018). Comparison of statin plus ezetimibe with double-dose statin on lipid profiles and inflammation markers. *Lipids in Health and Disease*.

[10] Naci H., Brugts J., Ades T. (2013). Comparative Tolerability and Harms of Individual Statins. *Circulation: Cardiovascular Quality and Outcomes*.

[11] Naci H., Brugts J. J., Fleurence R., Tsoi B., Toor H., Ades A. E. (2013). Comparative benefits of statins in the primary and secondary prevention of major coronary events and all-cause mortality: a network meta-analysis of placebo-controlled and active-comparator trials. *European Journal of Preventive Cardiology*.

[12] Naci H., Brugts J. J., Fleurence R., Ades A. E. (2013). Dose-comparative effects of different statins on serum lipid levels: a network meta-analysis of 256,827 individuals in 181 randomized controlled trials. *European Journal of Preventive Cardiology*.

[13] Hutton B., Salanti G., Caldwell D. M. (2015). The PRISMA extension statement for reporting of systematic reviews incorporating network meta-analyses of health care interventions: checklist and explanations. *Annals of Internal Medicine*.

[14] Comparative lipid-lowering efficacy and safety of 7 statins in patients with hyperlipidemia, hypercholesterolemia and diabetes mellitus: a network meta-analysis of randomized controlled trials. https://www.crd.york.ac.uk/prospero/display_record.php?ID=CRD42018108799.

[15] Chochrane Handbook Version 5.1.0., 8.5 The Cochrane Collaboration’s tool for assessing risk of bias. http://handbook-5-1.cochrane.org/.

[16] Cochrane Handbook Version 5.1.0., 16.1.3.2 Imputing standard deviations for changes from baseline, (2) Imputing a change-from-baseline standard deviation using a correlation coefficient. http://handbook-5-1.cochrane.org/.

[17] Mostafa S. A., Elrabat K., Mahrous M., Kamal M. (2018). Short term comparison between safety and efficacy of rosuvastatin 40 mg and atorvastatin 80 mg in patients with acute coronary syndrome. *Rational Pharmacotherapy in Cardiology*.

[18] Moezzi A., Reza Parizadeh S. M., Tavallaie S. (2014). Effects of simvastatin treatment on serum adiponectin concentrations in patients with dislipidemia. *Iran Red Crescent Med J*.

[19] Hernández C., Francisco G., Ciudin A. (2011). Effect of atorvastatin on lipoprotein (a) and interleukin-10: a randomized placebo-controlled trial. *Diabetes & Metabolism*.

[20] Durazzo A. E. S., Machado F. S., Ikeoka D. T. (2004). Reduction in cardiovascular events after vascular surgery with atorvastatin: a randomized trial. *Journal of Vascular Surgery*.

[21] Tan K. C. B., Chow W. S., Tam S. C. F., Ai V. H., Lam C. H., Lam K. S. (2002). Atorvastatin lowers C-reactive protein and improves endothelium-dependent vasodilation in type 2 diabetes mellitus. *The Journal of Clinical Endocrinology & Metabolism*.

[22] Wan X., Wang W., Liu J., Tong T. (2014). Estimating the sample mean and standard deviation from the sample size, median, range and/or interquartile range.. *BMC Medical Research Methodology*.

[23] Cochrane Handbook Version 5.1.0., 7.7.3.8. Combining Groups, formulae in Table 7.7.a. http://handbook-5-1.cochrane.org/.

[24] Zhao S., Peng D. (2017). Efficacy and safety of rosuvastatin versus atorvastatin in high-risk Chinese patients with hypercholesterolemia: a randomized, double blind, active-controlled study. *Current Medical Research and Opinion*.

[25] Ose L., Budinski D., Hounslow N., Arneson V. (2009). Comparison of pitavastatin with simvastatin in primary hypercholesterolaemia or combined dyslipidaemia. *Current Medical Research and Opinion*.

[26] Schwartz G. G., Bolognese M. A., Tremblay B. P. (2004). Efficacy and safety of rosuvastatin and atorvastatin in patients with hypercholesterolemia and a high risk of coronary heart disease: a randomized, controlled trial. *American Heart Journal*.

[27] Isaacsohn J., Hunninghake D., Schrott H. (2003). Effects of simvastatin, an HMG-CoA reductase inhibitor, in patients with hypertriglyceridemia. *Clinical Cardiology*.

[28] Mitropoulos K. A., Armitage J. M., Collins R. (1997). Randomized placebo-controlled study of the effects of simvastatin on haemostatic variables, lipoproteins and free fatty acids. The Oxford Cholesterol Study Group. *European Heart Journal*.

[29] White I. R. (2009). Multivariate random-effects meta-analysis. *The Stata Journal*.

[30] Higgins J. P. T., Jackson D., Barrett J. K., Lu G., Ades A. E., White I. R. (2012). Consistency and inconsistency in network meta-analysis: concepts and models for multi-arm studies. *Research Synthesis Methods*.

[31] White I. R. (2011). Multivariate random-effects meta-regression: updates to mvmeta. *The Stata Journal: Promoting communications on statistics and Stata*.

[32] White I. R., Barrett J. K., Jackson D., Higgins J. P. T. (2012). Consistency and inconsistency in network meta-analysis: model estimation using multivariate meta-regression. *Research Synthesis Methods*.

[33] White I. R. (2015). Software updates: st0156 2: multivariate random-effects meta-analysis. *The Stata Journal*.

[34] Dias S., Welton N. J., Caldwell D. M., Ades A. E. (2010). Checking consistency in mixed treatment comparison meta-analysis. *Statistics in Medicine*.

[35] Barkat M., Roy I., Antoniou S. A., Torella F., Antoniou G. A. (2018). Systematic review and network meta-analysis of treatment strategies for asymptomatic carotid disease. *Scientific Reports*.

[36] Zhu Y. C., Jiang X. Z., Bai Q. K. (2019). Evaluating the efficacy of atorvastatin on patients with carotid plaque by an innovative ultrasonography. *Journal of Stroke and Cerebrovascular Diseases*.

[37] Tunçez A., Altunkeser B. B., Öztürk B. (2019). Comparative effects of atorvastatin 80 mg and rosuvastatin 40 mg on the levels of serum endocan, chemerin, and galectin-3 in patients with acute myocardial infarction. *Anatolian Journal of Cardiology*.

[38] Thondapu V., Kurihara O., Yonetsu T. (2019). Comparison of rosuvastatin versus atorvastatin for coronary plaque stabilization. *The American Journal of Cardiology*.

[39] Canas J. A., Ross J. L., Taboada M. V. (2015). A randomized, double blind, placebo-controlled pilot trial of the safety and efficacy of atorvastatin in children with elevated low-density lipoprotein cholesterol (LDL-C) and type 1 diabetes. *Pediatric Diabetes*.

[40] Aydin M. U., Aygul N., Altunkeser B. B., Unlu A., TAner A. (2015). Comparative effects of high-dose atorvastatin versus moderate-dose rosuvastatin on lipid parameters, oxidized-LDL and inflammatory markers in ST elevation myocardial infarction. *Atherosclerosis*.

[41] Correa V., Fuchs F. D., Moreira L. B. (2014). Blood pressure-lowering effect of simvastatin: a placebo-controlled randomized clinical trial with 24-h ambulatory blood pressure monitoring. *Journal of Human Hypertension*.

[42] Koh K. K., Quon M. J., Sakuma I. (2013). Differential metabolic effects of rosuvastatin and pravastatin in hypercholesterolemic patients. *International Journal of Cardiology*.

[43] Nozue T., Hattori H., Ishihara M. (2013). Comparison of effects of pitavastatin versus pravastatin on serum proprotein convertase subtilisin/kexin type 9 levels in statin-naive patients with coronary artery disease. *The American Journal of Cardiology*.

[44] Nohara R., Daida H., Hata M. (2012). Effect of intensive lipid-lowering therapy with rosuvastatin on progression of carotid intima-media thickness in Japanese patients: Justification for Atherosclerosis Regression Treatment (JART) study. *Circulation Journal*.

[45] Lee C. W., Kang S. J., Ahn J. M. (2012). Comparison of effects of atorvastatin (20 mg) versus rosuvastatin (10 mg) therapy on mild coronary atherosclerotic plaques (from the ARTMAP Trial). *The American Journal of Cardiology*.

[46] Nicholls S. J., Ballantyne C. M., Barter P. J. (2011). Effect of two intensive statin regimens on progression of coronary disease. *The New England Journal of Medicine*.

[47] Saku K., Zhang B., Noda K. (2011). Randomized head-to-head comparison of pitavastatin, atorvastatin, and rosuvastatin for safety and efficacy (quantity and quality of LDL): -The PATROL Trial- (UMIN Registration No. 000000586). *Nihon Naika Gakkai Zasshi*.

[48] Tsutamoto T., Sakai H., Ibe K. (2011). Effect of atorvastatin vs. rosuvastatin on cardiac sympathetic nerve activity in non-diabetic patients with dilated cardiomyopathy. *Circulation Journal*.

[49] Shimabukuro M., Higa M., Tanaka H., Shimabukuro T., Yamakawa K., Masuzaki H. (2011). Distinct effects of pitavastatin and atorvastatin on lipoprotein subclasses in patients with type 2 diabetes mellitus. *Diabetic Medicine*.

[50] Heart Protection Study Collaborative Group (2011). Effects on 11-year mortality and morbidity of lowering LDL cholesterol with simvastatin for about 5 years in 20 536 high-risk individuals: a randomised controlled trial. *The Lancet*.

[51] Sansanayudh N., Wongwiwatthananukit S., Putwai P., Dhumma-Upakorn R. (2010). Comparative efficacy and safety of low-dose pitavastatin versus atorvastatin in patients with hypercholesterolemia. *The Annals of Pharmacotherapy*.

[52] Bellia A., Rizza S., Galli A. (2010). Early vascular and metabolic effects of rosuvastatin compared with simvastatin in patients with type 2 diabetes. *Atherosclerosis*.

[53] Su Y., Xu Y., Sun Y. M. (2010). Comparison of the effects of simvastatin versus atorvastatin on oxidative stress in patients with type 2 diabetes mellitus. *Journal of Cardiovascular Pharmacology*.

[54] Kurabayashi M., Yamazaki T., SUBARU Study Group (2008). Superior benefit of aggressive lipid-lowering therapy for high-risk patients using statins: the SUBARU Study -more hypercholesterolemic patients achieve Japan Atherosclerosis Society LDL-C goals with rosuvastatin therapy than with atorvastatin therapy. *Journal of Atherosclerosis and Thrombosis*.

[55] Hong Y. J., Jeong M. H., Chung J. W. (2008). The effects of rosuvastatin on plaque regression in patients who have a mild to moderate degree of coronary stenosis with vulnerable plaque. *Korean Circulation Journal*.

[56] Yun K. H., Park H.-Y., Choi J.-H. (2007). Comparison of efficacy and safety after administering high potency statin to high risk patients: rosuvastatin 10 mg versus atorvastatin 20 mg. *Korean Circulation Journal*.

[57] Kom G. D., Schwedhelm E., Maas R., Schneider L., Benndorf R., Böger R. H. (2007). Impact of atorvastatin treatment on platelet-activating factor acetylhydrolase and 15-F2trans-isoprostane in hypercholesterolaemic patients. *British Journal of Clinical Pharmacology*.

[58] Marketou M. E., Zacharis E. A., Nikitovic D. (2006). Early effects of simvastatin versus atorvastatin on oxidative stress and proinflammatory cytokines in hyperlipidemic subjects. *Angiology*.

[59] Pedersen T. R., Faergeman O., Kastelein J. J. (2005). High-dose atorvastatin vs usual-dose simvastatin for secondary prevention after myocardial infarction: The IDEAL study: a randomized controlled trial. *JAMA*.

[60] Sirtori C. R., Calabresi L., Pisciotta L. (2005). Effect of statins on LDL particle size in patients with familial combined hyperlipidemia: a comparison between atorvastatin and pravastatin. *Nutrition, Metabolism and Cardiovascular Diseases*.

[61] Nissen S. E., Tuzcu E. M., Schoenhagen P. (2004). Effect of intensive compared with moderate lipid-lowering therapy on progression of coronary atherosclerosis : a randomized controlled trial. *JAMA*.

[62] Bevilacqua M., Guazzini B., Righini V., Barrella M., Toscano R., Chebat E. (2004). Metabolic effects of fluvastatin extended release 80 mg and atorvastatin 20 mg in patients with type 2 diabetes mellitus and low serum high-density lipoprotein cholesterol levels: a 4-month, prospective, open-label, randomized, blinded—end point (probe) trial. *Current Therapeutic Research, Clinical and Experimental*.

[63] Colhoun H. M., Betteridge D. J., Durrington P. N. (2004). Primary prevention of cardiovascular disease with atorvastatin in type 2 diabetes in the Collaborative Atorvastatin Diabetes Study (CARDS): multicentre randomised placebo-controlled trial. *Lancet*.

[64] van Wissen S., Smilde T. J., Trip M. D., de Boo T., Kastelein J. J., Stalenhoef A. F. (2003). Long term statin treatment reduces lipoprotein(a) concentrations in heterozygous familial hypercholesterolaemia. *Heart*.

[65] Mccrindle B. W., Ose L., Marais A. D. (2003). Efficacy and safety of atorvastatin in children and adolescents with familial hypercholesterolemia or severe hyperlipidemia: a multicenter, randomized, placebo-controlled trial. *The Journal of Pediatrics*.

[66] Kadikoylu G., Yukselen V., Yavasoglu I., Bolaman Z. (2003). Hemostatic effects of atorvastatin versus simvastatin. *The Annals of Pharmacotherapy*.

[67] Manuel-Y-Keenoy B., Van Campenhout C., Vertommen J., De Leeuw I. (2003). Effects of atorvastatin on LDL sub-fractions and peroxidation in type 1 diabetic patients: a randomised double-blind placebo-controlled study. *Diabetes/Metabolism Research and Reviews*.

[68] Kübler W. (2003). Prevention of coronary and stroke events with atorvastatin in hypertensive patients who have average or lower-than-average cholesteroal concentrations, in the Anglo-Scandinavian Cardiac Outcomes Trial—Lipid Lowering Arm (ASCOL-LLA): a multicentre randomised controlled trial. *Drugs*.

[69] Winkler K., Abletshauser C., Hoffmann M. M. (2002). Effect of fluvastatin slow-release on low density lipoprotein (LDL) subfractions in patients with type 2 diabetes mellitus: baseline LDL profile determines specific mode of action. *The Journal of Clinical Endocrinology & Metabolism*.

[70] Wang K.-Y., Ting C.-T. (2001). A Randomized, Double-blind, Placebo-controlled, 8-week Study to Evaluate the Efficacy and Safety of Once Daily Atrovastatin (10 mg) in Patients with Elevated LDL-cholesterol. *Japanese Heart Journal*.

[71] Schrott H. G., Knapp H., Davila M., Shurzinske L., Black D. (2000). Effect of atorvastatin on blood lipid levels in the first 2 weeks of treatment: a randomized, placebo-controlled study. *American Heart Journal*.

[72] Serruys P. W., Foley D. P., Jackson G. (1999). A randomized placebo-controlled trial of fluvastatin for prevention of restenosis after successful coronary balloon angioplasty Final results of the fluvastatin angiographic restenosis (FLARE) trial. *European Heart Journal*.

[73] Lam K. S., Cheng I. K., Janus E. D., Pang R. W. (1995). Cholesterol-lowering therapy may retard the progression of diabetic nephropathy. *Diabetologia*.

[74] Contermans J., Smit J. W., Bar P. R., Erkelens D. W. (1995). A comparison of the effects of simvastatin and pravastatin monotherapy on muscle histology and permeability in hypercholesterolaemic patients. *British Journal of Clinical Pharmacology*.

[75] McDowell I. F., Smye M., Trinick T. (1991). Simvastatin in severe hypercholesterolaemia: a placebo controlled trial. *British Journal of Clinical Pharmacology*.

[76] Tonelli M., Lloyd A., Clement F. (2011). Efficacy of statins for primary prevention in people at low cardiovascular risk: a meta-analysis. *Canadian Medical Association Journal*.

[77] Kapur N., Musunuru K. (2008). Clinical efficacy and safety of statins in managing cardiovascular risk. *Vascular Health and Risk Management*.

