# Supplementary material for: Comparative Lipid-Lowering/Increasing Efficacy of 7 Statins in Patients with Dyslipidemia, Cardiovascular Diseases, or Diabetes Mellitus: Systematic Review and Network Meta-Analyses of 50 Randomized Controlled Trials
Source: Cardiovasc Ther. 2020 Apr 23;2020:3987065. doi: 10.1155/2020/3987065 (PMC7201823; doi:10.1155/2020/3987065)
Supplement: Supplementary Materials — The Supplementary Material consists of 4 parts. Appendix S1: the complete literature search strategy of the network meta-analysis (NMA). Appendix S2: description of the unit conversion methods used in this study. Appendix S3: league tables of the NMAs conducted for 5 of the outcomes. Appendix S4: the PRISMA checklist of the NMA. [file 3987065.f1.pdf]

Appendix S1

The complete literature search strategy of the network meta-analysis and the number of literatures firstly retrieved:

PubMed

((((((((lovastatin) OR pravastatin) OR simvastatin) OR fluvastatin) OR atorvastatin) OR rosuvastatin) OR pitavastatin) OR statins) AND randomized controlled trial

7377

Embase

(lovastatin OR pravastatin OR simvastatin OR fluvastatin OR atorvastatin OR rosuvastatin OR pitavastatin OR statins) AND randomized AND controlled AND trial

10114

Web of Science

(((((((TITLE: (lovastatin) OR TITLE: (pravastatin)) OR TITLE: (simvastatin)) OR TITLE: (fluvastatin)) OR TITLE: (atorvastatin)) OR TITLE: (rosuvastatin)) OR TITLE: (pitavastatin)) OR (TITLE: (statins) AND TOPIC: (randomized controlled trial)))

667

Cochrane

All Text: lovastatin OR pravastatin OR simvastatin OR fluvastatin OR atorvastatin OR rosuvastatin OR pivastatin OR statins AND randomized controlled trial in Trials (Word variations have been searched)

9004

Appendix S2

The unit conversion methods used in this study are shown as follows:

Outcome	equation	Conversion coefficient
LDL-C	$1mmol \cdot L^{-1} = 38.61\ mg \cdot dl^{-1}$	38.61
HDL-C	$1mmol \cdot L^{-1} = 38.76\ mg \cdot dl^{-1}$	38.76
TC	$1mmol \cdot L^{-1} = 38.76\ mg \cdot dl^{-1}$	38.76
TG	$1mmol \cdot L^{-1} = 88.50\ mg \cdot dl^{-1}$	88.50
ApoB	Not applicable, didn't use unit conversion.	
ApoA1	Not applicable, didn't use unit conversion.	

Appendix S3

Table S3-1. League table of the HDL-C network meta-analysis results among placebo and 7 statins.(Results with statistical significance are shown in bold.)

	Fluvastatin	_Lovastatin_	_Pravastatin_	_Pitavastatin_	_Atorvastatin_	_Simvastatin_	_Rosuvastatin	_Placebo_
Fluvastatin	Fluvastatin	-0.91 (-12.54, 10.72)	-1.93 (-10.22, 6.37)	-1.91 (-10.65, 6.83)	-2.99 (-10.03, 4.06)	-3.16 (-10.65, 4.34)	-4.12 (-11.63, 3.39)	-4.78 (-11.89, 2.32)
Lovastatin	0.91 (-10.72, 12.54)	Lovastatin	-1.02 (-11.52, 9.49)	-1.00 (-11.91, 9.92)	-2.08 (-11.78, 7.62)	-2.24 (-12.05, 7.57)	-3.21 (-13.18, 6.77)	-3.87 (-13.08, 5.34)
Pravastatin	1.93 (-6.37, 10.22)	1.02 (-9.49, 11.52)	Pravastatin	0.02 (-6.06,6.10)	-1.06 (-5.69, 3.57)	-1.23 (-6.33,3.88)	-2.19 (-6.95, 2.56)	-2.85 (-7.91, 2.20)
Pitavastatin	1.91 (-6.83, 10.65)	1.00 (-9.92, 11.91)	-0.02 (-6.10, 6.06)	Pitavastatin	-1.08 (-6.39, 4.22)	-1.25 (-6.98, 4.49)	-2.21 (-7.78, 3.36)	-2.87 (-8.72, 2.97)
Atorvastatin	2.99 (-4.06, 10.03)	2.08 (-7.62, 11.78)	1.06 (-3.57,5.69)	1.08 (-4.22,6.39)	Atorvastatin	-0.17 (-3.38, 3.04)	-1.13 (-3.92, 1.65)	-1.79 (-4.83, 1.24)
Simvastatin	3.16 (-4.34, 10.65)	2.24 (-7.57, 12.05)	1.23 (-3.88,6.33)	1.25 (-4.49,6.98)	0.17 (-3.04,3.38)	Simvastatin	-0.96 (-4.90, 2.97)	-1.63 (-5.00, 1.75)
Rosuvastatin	4.12 (-3.39, 11.63)	3.21 (-6.77, 13.18)	2.19 (-2.56,6.95)	2.21 (-3.36,7.78)	1.13 (-1.65,3.92)	0.96 (-2.97,4.90)	Rosuvastatin	-0.66 (-4.49, 3.16)
Placebo	4.78 (-2.32, 11.89)	3.87 (-5.34, 13.08)	2.85 (-2.20,7.91)	2.87 (-2.97,8.72)	1.79 (-1.24,4.83)	1.63 (-1.75, 5.00)	0.66 (-3.16,4.49)	Placebo

Table S3-2. League table of the TC network meta-analysis results among placebo and 7 statins. (Results with statistical significance are shown in bold.)

	Lovastatin	_Atorvastatin_	_Rosuvastatin_	_Simvastatin_	_Pitavastatin_	_Fluvastatin_	_Pravastatin_	_Placebo_
Lovastatin	Lovastatin	**183.69 (122.83,244.56)**	**183.81 (120.79,246.83)**	**195.36 (133.80,256.92)**	**200.63 (130.62, 270.64)**	**210.47 (126.16, 294.78)**	**217.11 (150.12,284.11)**	**267.44 (208.87, 326.02)**
Atorvastatin	**-183.69 (-244.56, -122.83)**	Atorvastatin	0.12 (-18.40,18.64)	11.67 (-7.57, 30.91)	16.94 (-19.97,53.84)	26.77 (-36.09, 89.64)	**33.42 (2.11, 64.74)**	**83.75 (67.20, 100.31)**
Rosuvastatin	**-183.81 (-246.83, -120.79)**	-0.12 (-18.64, 18.40)	Rosuvastatin	11.55 (-13.04, 36.14)	16.82 (-23.34, 56.97)	26.66 (-38.29, 91.61)	33.30 (-0.70, 67.31)	**83.63 (60.37, 106.89)**
Simvastatin	**-195.36 (-256.92, -133.80)**	-11.67 (-30.91, 7.57)	-11.55 (-36.14, 13.04)	Simvastatin	5.27 (-31.88, 42.43)	15.11 (-48.42, 78.64)	21.76 (-10.74, 54.25)	**72.08 (53.15, 91.02)**
Pitavastatin	**-200.63 (-270.64, -130.62)**	-16.94 (-53.84, 19.97)	-16.82 (-56.97, 23.34)	-5.27 (-42.43, 31.88)	Pitavastatin	9.84 (-61.91, 81.59)	16.49 (-23.43, 56.40)	**66.81 (28.47, 105.16)**
Fluvastatin	**-210.47 (-294.78 ,-126.16)**	-26.77 (-89.64, 36.09)	-26.66 (-91.61, 38.29)	-15.11 (-78.64, 48.42)	-9.84 (-81.59, 61.91)	Fluvastatin	6.65 (-62.16, 75.46)	56.98 (-3.67, 117.62)
Pravastatin	**-217.11 (-284.11, -150.12)**	**-33.42 (-64.74, -2.11)**	-33.30 (-67.31, 0.70)	-21.76 (-54.25, 10.74)	-16.49 (-56.40, 23.43)	-6.65 (-75.46, 62.16)	Pravastatin	**50.33 (17.81, 82.84)**
Placebo	**-267.44 (-326.02, -208.87)**	**-83.75 (-100.31, -67.20)**	**-83.63 (-106.89, -60.37)**	**-72.08 (-91.02, -53.15)**	**-66.81 (-105.16, -28.47)**	-56.98 (-117.62, 3.67)	**-50.33 (-82.84,-17.81)**	Placebo

Table S3-3. League table of the TG network meta-analysis results among placebo and 7 statins. (Results with statistical significance are shown in bold.)

	Lovastatin	_Pravastatin_	_Fluvastatin_	_Rosuvastatin_	_Atorvastatin_	_Simvastatin_	_Pitavastatin_	_Placebo_
Lovastatin	Lovastatin	48.03 (-19.68, 115.73)	47.81 (-33.97, 129.58)	56.32 (-8.86, 121.51)	58.04 (-5.36, 121.43)	64.35 (-0.28, 128.99)	66.91 (-2.08, 135.90)	**88.50 (27.70,149.30)**
Pravastatin	-48.03 (-115.73, 19.68)	Pravastatin	-0.22 (-60.90, 60.46)	8.30 (-19.77, 36.36)	10.01 (-17.22, 37.24)	16.33 (-13.18, 45.84)	18.88 (-15.33, 53.10)	**40.47 (10.68,70.26)**
Fluvastatin	-47.81 (-129.58, 33.97)	0.22 (-60.46, 60.90)	Fluvastatin	8.52 (-49.03, 66.06)	10.23 (-44.91, 65.38)	16.55 (-40.78, 73.88)	19.11 (-42.77, 80.98)	40.69 (-13.99, 95.38)
Rosuvastatin	-56.32 (-121.51, 8.86)	-8.30 (-36.36, 19.77)	-8.52 (-66.06, 49.03)	Rosuvastatin	1.72 (-16.34, 19.78)	8.03 (-15.62, 31.68)	10.59 (-20.69, 41.87)	**32.18 (8.67, 55.69)**
Atorvastatin	-58.04 (-121.43, 5.36)	-10.01 (-37.24, 17.22)	-10.23 (-65.38, 44.91)	-1.72 (-19.78, 16.34)	Atorvastatin	6.32 (-12.97, 25.60)	8.87 (-20.23, 37.98)	**30.46 (12.51, 48.41)**
Simvastatin	-64.35 (-128.99, 0.28)	-16.33 (-45.84, 13.18)	-16.55 (-73.88, 40.78)	-8.03 (-31.68, 15.62)	-6.32 (-25.60, 12.97)	Simvastatin	2.56 (-28.44, 33.55)	**24.15 (2.20, 46.09)**
Pitavastatin	-66.91 (-135.90, 2.08)	-18.88 (-53.10, 15.33)	-19.11 (-80.98, 42.77)	-10.59 (-41.87, 20.69)	-8.87 (-37.98, 20.23)	-2.56 (-33.55, 28.44)	Pitavastatin	21.59 (-11.01, 54.19)
Placebo	**-88.50 (-149.30, -27.70)**	**-40.47 (-70.26, -10.68)**	-40.69 (-95.38, 13.99)	**-32.18 (-55.69, -8.67)**	**-30.46 (-48.41, -12.51)**	**-24.15 (-46.09, -2.20)**	-21.59 (-54.19, 11.01)	Placebo

Table S3-4. League table of the ApoA1 network meta-analysis results among placebo and 7 statins. (Results with statistical significance are shown in bold.)

	Rosuvastatin	_Simvastatin_	_Fluvastatin_	_Atorvastatin_	_Lovastatin_	_Placebo_	_Pitavastatin_	_Pravastatin_
Rosuvastatin	Rosuvastatin	-0.60 (-7.36,6.16)	-1.25 (-9.05,6.54)	-2.63 (-6.57,1.32)	-4.53 (-12.79, 3.73)	**-4.62 (-9.24, -0.01)**	-8.31 (-21.42, 4.80)	**-7.31 (-13.75, -0.87)**
Simvastatin	0.60 (-6.16,7.36)	Simvastatin	-0.65 (-8.67,7.36)	-2.02 (-7.55,3.50)	-3.93 (-12.82, 4.96)	-4.02 (-9.69,1.65)	-7.71 (-21.85, 6.44)	-6.71 (-15.06, 1.64)
Fluvastatin	1.25 (-6.54,9.05)	0.65 (-7.36,8.67)	Fluvastatin	-1.37 (-7.97,5.23)	-3.28 (-12.48, 5.92)	-3.37 (-9.51,2.77)	-7.06 (-21.56, 7.45)	-6.06 (-15.00, 2.89)
Atorvastatin	2.63 (-1.32,6.57)	2.02 (-3.50,7.55)	1.37 (-5.23,7.97)	Atorvastatin	-1.91 (-9.49,5.68)	-2.00 (-5.26,1.27)	-5.68 (-18.89, 7.52)	-4.68 (-11.31, 1.94)
Lovastatin	4.53 (-3.73,12.79)	3.93 (-4.96,12.82)	3.28 (-5.92,12.48)	1.91 (-5.68,9.49)	Lovastatin	-0.09 (-6.94,6.76)	-3.78 (-18.57, 11.01)	-2.78 (-12.18, 6.62)
Placebo	**4.62 (0.01,9.24)**	4.02 (-1.65,9.69)	3.37 (-2.77,9.51)	2.00 (-1.27,5.26)	0.09 (-6.76,6.94)	Placebo	-3.69 (-16.80, 9.42)	-2.69 (-9.13, 3.75)
Pitavastatin	8.31 (-4.80,21.42)	7.71 (-6.44,21.85)	7.06 (-7.45,21.56)	5.68 (-7.52,18.89)	3.78 (-11.01, 18.57)	3.69 (-9.42,16.80)	Pitavastatin	1.00 (-10.42, 12.42)
Pravastatin	**7.31 (0.87,13.75)**	6.71 (-1.64,15.06)	6.06 (-2.89,15.00)	4.68 (-1.94,11.31)	2.78 (-6.62,12.18)	2.69 (-3.75,9.13)	-1.00 (-12.42, 10.42)	Pravastatin

Table S3-5. League table of the ApoB network meta-analysis results among placebo and 7 statins. (Results with statistical significance are shown in bold.)

	Rosuvastatin	_Simvastatin_	_Atorvastatin_	_Pitavastatin_	_Pravastatin_	_Fluvastatin_	_Lovastatin_	_Placebo_
Rosuvastatin	Rosuvastatin	2.35 (-41.09, 45.80)	4.37 (-16.62, 25.35)	1.40 (-60.98, 63.78)	7.40 (-34.26, 49.06)	18.67 (-20.33, 57.68)	35.38 (-17.16, 87.92)	**35.80 (9.89, 61.71)**
Simvastatin	-2.35 (-45.80, 41.09)	Simvastatin	2.02 (-36.65, 40.68)	-0.95 (-73.92, 72.02)	5.05 (-51.24, 61.34)	16.32 (-34.04, 66.69)	33.03 (-28.11, 94.17)	33.45 (-7.16, 74.06)
Atorvastatin	-4.37 (-25.35, 16.62)	-2.02 (-40.68, 36.65)	Atorvastatin	-2.97 (-65.98, 60.05)	3.03 (-39.57, 45.63)	14.30 (-19.77, 48.38)	31.01 (-18.77, 80.79)	**31.43 (11.70, 51.16)**
Pitavastatin	-1.40 (-63.78, 60.98)	0.95 (-72.02, 73.92)	2.97 (-60.05, 65.98)	Pitavastatin	6.00 (-40.44, 52.43)	17.27 (-52.69, 87.23)	33.98 (-43.36, 111.32)	34.40 (-27.99, 96.78)
Pravastatin	-7.40 (-49.06, 34.26)	-5.05 (-61.34, 51.24)	-3.03 (-45.63, 39.57)	-6.00 (-52.43, 40.44)	Pravastatin	11.27 (-41.06, 63.60)	27.98 (-33.86, 89.82)	28.40 (-13.26, 70.06)
Fluvastatin	-18.67 (-57.68, 20.33)	-16.32 (-66.69, 34.04)	-14.30 (-48.38, 19.77)	-17.27 (-87.23, 52.69)	-11.27 (-63.60, 41.06)	Fluvastatin	16.71 (-40.26, 73.67)	17.13 (-16.87, 51.12)
Lovastatin	-35.38 (-87.92, 17.16)	-33.03 (-94.17, 28.11)	-31.01 (-80.79, 18.77)	-33.98 (-111.32, 43.36)	-27.98 (-89.82, 33.86)	-16.71 (-73.67, 40.26)	Lovastatin	0.42 (-45.29, 46.13)
Placebo	**-35.80 (-61.71, -9.89)**	-33.45 (-74.06, 7.16)	**-31.43 (-51.16, -11.70)**	-34.40 (-96.78, 27.99)	-28.40 (-70.06, 13.26)	-17.13 (-51.12, 16.87)	-0.42 (-46.13, 45.29)	Placebo

Appendix S4 PRISMA Checklist

Section/topic	#	Checklist item	Reported on page #
**TITLE**			
Title	1	Identify the report as a systematic review, meta-analysis, or both.	1
**ABSTRACT**			
Structured summary	2	Provide a structured summary including, as applicable: background; objectives; data sources; study eligibility criteria, participants, and interventions; study appraisal and synthesis methods; results; limitations; conclusions and implications of key findings; systematic review registration number.	2
**INTRODUCTION**			
Rationale	3	Describe the rationale for the review in the context of what is already known.	3
Objectives	4	Provide an explicit statement of questions being addressed with reference to participants, interventions, comparisons, outcomes, and study design (PICOS).	4
**METHODS**			
Protocol and registration	5	Indicate if a review protocol exists, if and where it can be accessed (e.g., Web address), and, if available, provide registration information including registration number.	4
Eligibility criteria	6	Specify study characteristics (e.g., PICOS, length of follow-up) and report characteristics (e.g., years considered, language, publication status) used as criteria for eligibility, giving rationale.	5
Information sources	7	Describe all information sources (e.g., databases with dates of coverage, contact with study authors to identify additional studies) in the search and date last searched.	4
Search	8	Present full electronic search strategy for at least one database, including any limits used, such that it could be repeated.	4 & Appendix S1
Study selection	9	State the process for selecting studies (i.e., screening, eligibility, included in systematic review, and, if applicable, included in the meta-analysis).	5
Data collection process	10	Describe method of data extraction from reports (e.g., piloted forms, independently, in duplicate) and any processes for obtaining and confirming data from investigators.	5-6

Data items	11	List and define all variables for which data were sought (e.g., PICOS, funding sources) and any assumptions and simplifications made.	5-6
Risk of bias in individual studies	12	Describe methods used for assessing risk of bias of individual studies (including specification of whether this was done at the study or outcome level), and how this information is to be used in any data synthesis.	5
Summary measures	13	State the principal summary measures (e.g., risk ratio, difference in means).	5
Synthesis of results	14	Describe the methods of handling data and combining results of studies, if done, including measures of consistency (e.g., I^2) for each meta-analysis.	5-6

Section/topic	#	Checklist item	Reported on page #
Risk of bias across studies	15	Specify any assessment of risk of bias that may affect the cumulative evidence (e.g., publication bias, selective reporting within studies).	21,22
Additional analyses	16	Describe methods of additional analyses (e.g., sensitivity or subgroup analyses, meta-regression), if done, indicating which were pre-specified.	6,28-29
**RESULTS**			
Study selection	17	Give numbers of studies screened, assessed for eligibility, and included in the review, with reasons for exclusions at each stage, ideally with a flow diagram.	8
Study characteristics	18	For each study, present characteristics for which data were extracted (e.g., study size, PICOS, follow-up period) and provide the citations.	9-14
Risk of bias within studies	19	Present data on risk of bias of each study and, if available, any outcome level assessment (see item 12).	21,22
Results of individual studies	20	For all outcomes considered (benefits or harms), present, for each study: (a) simple summary data for each intervention group (b) effect estimates and confidence intervals, ideally with a forest plot.	27,Appendix S3
Synthesis of results	21	Present results of each meta-analysis done, including confidence intervals and measures of consistency.	23-27
Risk of bias across studies	22	Present results of any assessment of risk of bias across studies (see Item 15).	21-22
Additional analysis	23	Give results of additional analyses, if done (e.g., sensitivity or subgroup analyses, meta-regression [see Item 16]).	28-30
**DISCUSSION**			
Summary of evidence	24	Summarize the main findings including the strength of evidence for each main outcome; consider their relevance	23-25

		to key groups (e.g., healthcare providers, users, and policy makers).	
Limitations	25	Discuss limitations at study and outcome level (e.g., risk of bias), and at review-level (e.g., incomplete retrieval of identified research, reporting bias).	32
Conclusions	26	Provide a general interpretation of the results in the context of other evidence, and implications for future research.	32
**FUNDING**			
Funding	27	Describe sources of funding for the systematic review and other support (e.g., supply of data); role of funders for the systematic review.	32-33

From: Moher D, Liberati A, Tetzlaff J, Altman DG, The PRISMA Group (2009). Preferred Reporting Items for Systematic Reviews and Meta-Analyses: The PRISMA Statement. PLoS Med 6(7): e1000097. doi:10.1371/journal.pmed1000097
